# Na_2_CO_3_*-*responsive mechanisms in halophyte *Puccinellia tenuiflora* roots revealed by physiological and proteomic analyses

**DOI:** 10.1038/srep32717

**Published:** 2016-09-06

**Authors:** Qi Zhao, Jinwei Suo, Sixue Chen, Yudan Jin, Xiaolin Ma, Zepeng Yin, Yuhong Zhang, Tai Wang, Ji Luo, Wenhai Jin, Xia Zhang, Zhiqiang Zhou, Shaojun Dai

**Affiliations:** 1 Key Laboratory of Forest Plant Ecology, Ministry of Education, Northeast Forestry University, Harbin 150040, China; 2 Alkali Soil Natural Environmental Science Center, Northeast Forestry University, Key Laboratory of Saline-alkali Vegetation Ecology Restoration in Oil Field, Ministry of Education, Harbin 150040, China; 3 Department of Biology, Genetics Institute, Plant Molecular and Cellular Biology Program, Interdisciplinary Center for Biotechnology Research, University of Florida, Gainesville, FL 32610, USA; 4 Institute of Botany, Chinese Academy of Sciences, Beijing 100093, China; 5 AB Sciex Asia Pacific Application Support Center, Shanghai 200233, China

## Abstract

Soil alkalization severely affects crop growth and agricultural productivity. Alkali salts impose ionic, osmotic, and high pH stresses on plants. The alkali tolerance molecular mechanism in roots from halophyte *Puccinellia tenuiflora* is still unclear. Here, the changes associated with Na_2_CO_3_ tolerance in *P. tenuiflora* roots were assessed using physiological and iTRAQ-based quantitative proteomic analyses. We set up the first protein dataset in *P. tenuiflora* roots containing 2,671 non-redundant proteins. Our results showed that Na_2_CO_3_ slightly inhibited root growth, caused ROS accumulation, cell membrane damage, and ion imbalance, as well as reduction of transport and protein synthesis/turnover. The Na_2_CO_3_-responsive patterns of 72 proteins highlighted specific signaling and metabolic pathways in roots. Ca^2+^ signaling was activated to transmit alkali stress signals as inferred by the accumulation of calcium-binding proteins. Additionally, the activities of peroxidase and glutathione peroxidase, and the peroxiredoxin abundance were increased for ROS scavenging. Furthermore, ion toxicity was relieved through Na^+^ influx restriction and compartmentalization, and osmotic homeostasis reestablishment due to glycine betaine accumulation. Importantly, two transcription factors were increased for regulating specific alkali-responsive gene expression. Carbohydrate metabolism-related enzymes were increased for providing energy and carbon skeletons for cellular metabolism. All these provide new insights into alkali-tolerant mechanisms in roots.

Soil alkalization is a major abiotic stress that severely affects crop growth and agricultural productivity worldwide. The alkaline soil contains high levels of Na_2_CO_3_ and NaHCO_3_, which leads to a high soil pH (>9.0)^1^. Relative to neutral salts, alkali salts impose more severe damage to plants due to the combination of ion toxicity, osmotic stress, and high pH stress. Especially, high pH environment surrounding the plant roots has great influence on nutrient uptake, organic acid balance, ion homeostasis, and especially pH stability at cell, tissue, and organ levels^2^,^3^,^4^.

Plant roots act as the primary site for perceiving the alkali stress. Alkaline soil always contains mixed saline-alkali, including NaCl, Na_2_CO_3_, NaHCO_3_, Na_2_SO_4_, and NaOH, which generally retards the root growth and even kills the plants^5^. A mixed saline-alkali (70 mM NaCl and 50 mM NaHCO_3_) stress activated a series of signaling and metabolic pathways in roots of glycophyte soybean (*Glycine max*), including hormone signaling, transcriptional regulation, ion homeostasis, antioxidant responses, transportation, protein synthesis and destination, cell rescue and defense^6^. Importantly, single alkali stress also has obvious effect on root growth. In the Na_2_CO_3_-stressed roots of glycophyte sunflower (*Helianthus annuus*), cellular homeostasis was disrupted due to the increased Na^+^ content, decreased K^+^ content and protein concentration^7^. While the increased content of free amino acid and the enhanced activities of ATPase and proteases in roots suggest that the osmotic homeostasis, energy supply, and protein turnover play crucial roles in alkali tolerance^7^.

The neutral salt-responsive physiological and molecular mechanisms in roots have been extensively studied^8^,^9^,^10^, but the specific molecular mechanism underlying alkali tolerance in roots is lacking. A number of genes in roots were affected by alkali stress. It was reported that 8,319 genes, representing over a quarter of the total number in the maize (*Zea mays*) genome, were significantly altered in roots under 50 mM Na_2_CO_3_ treatment for 5 h^11^. The expression patterns of these alkali-responsive genes reveal that maize roots possess unique biological pathways for adapting to Na_2_CO_3_ stress. They include Na_2_CO_3_-induced brassinosteroid biosynthesis, and Na_2_CO_3_-reduced ascorbate (AsA) and aldarate metabolism, protein processing in endoplasmic reticulum (ER), the biosynthesis of N-glycan and fatty acid, and circadian rhythm^11^.

The early and delayed alkali-responsive functional inclines are different in roots revealed from high-throughput transcriptomic analysis. The patterns of 7,088 differentially expressed genes in wild soybean (*Glycine soja*) under 50 mM NaHCO_3_ stress showed that at early stage of stress (3–6 h), a cascade of processes were initiated, including the induced signal transduction, secondary metabolism, and transcription regulation, but these molecular processes were reduced after 12 h of stress, following the subsequent induction of protein synthesis and energy metabolism after 24 h of stress^12^. In addition, the NaHCO_3_-responsive genes in roots of woody halophyte *Tamarix hispida* under 300 mM NaHCO_3_ for 12 h, 24 h, and 48 h imply that various specific strategies are employed for surviving from alkaline stress, such as induced biosynthesis of proline and trehalose, enhancement of protein folding and osmotic homeostasis, and diverse transcription regulations^13^.

Although a large amount of candidate alkali-responsive genes were found using transcriptomic approaches, only several of them have been cloned and characterized. It was reported that three genes, including *rHsp90* (encoding a 90 kDa heat shock protein (Hsp))^14^, *RMtATP6* (encoding a mitochondrial ATP synthase 6 kDa subunit)^1^, and *NADP-ME*_*2*_ (encoding an NADP-malic enzyme)^15^, were isolated from a cDNA library constructed from rice (*Oryza sativa*) roots under NaHCO_3_ stress. The expression levels of these genes and the activity of NADP-malic enzyme were increased in rice roots under NaHCO_3_ and Na_2_CO_3_ treatments^1^,^14^,^15^. Moreover, yeast or transgenic plants over-expressing these genes exhibited greater tolerance to alkali/salt stress, indicating their important roles in alkali/salt tolerance^1^,^14^,^15^. However, these results are not adequate for unraveling the molecular basis and dynamic networks underlying alkaline tolerance in plant roots.

*Puccinellia tenuiflora* is a monocotyledonous halophyte widely distributed in the Songnen Plain in Northeastern China. *P. tenuiflora* belongs to the genus Gramineae, and has close genetic relationships with rice and barley (*Hordeum vulgare*)^16^,^17^. Unlike these two relatives, *P. tenuiflora* has a strong ability of salt and alkali tolerance to grow normally under maximum stress up to 600 mM NaCl and 150 mM Na_2_CO_3_ (pH 11.0) for 6 days^17^. Therefore, *P. tenuiflora* is considered as an outstanding pasture for soil improvement, as well as a good plant model among monocotyledonous plants for understanding alkali tolerance mechanisms.

The salt/alkali tolerance of *P. tenuiflora* was due to its high selectivity for K^+^ over Na^+ ^2,18. The low net Na^+^ uptake was mainly resulted from the restriction of unidirectional Na^+^ influx^2^. In addition, the Casparian band in the root endodermis can also block the apoplastic path of Na^+^ entrance^18^. Genes encoding several plasma membrane (PM) located proteins have been characterized to be involved in transmembrane ion transport, such as *PutPMP3-1* and *PutPMP3-2* encoding PM protein 3 family proteins function to prevent the accumulation of excess Na^+ ^19, *PutHKT2; 1* encoding a high-affinity K^+^ transporter which plays a role in K^+^ uptake to maintain a high ratio of K^+^/Na^+^ in the cells^20^, *PtNHA1* encoding a Na^+^/H^+^ antiporter for the maintenance of low cytosolic Na^+ ^21, and *PutAKT1* encoding a PM-localized K^+^ channel family protein that can interact with KPutB1 to alter K^+^ and Na^+^ homeostasis^22^. Besides of restriction of Na^+^ entrance, Na^+^ can also be secreted onto leaf surface through stomata or together with wax secretion under salt/alkali stress^23^,^24^,^25^. However, the Na^+^ secretion accounted for only a small portion of the whole plant Na^+^ content and was very small compared with other salt-secreting halophytes^2^.

To maintain intracellular ionic and osmotic balance under saline or alkaline stress, *P. tenuiflora* can accumulate organic acids and inorganic anions to balance the massive influx of cations^4^,^24^. Additionally, *P. tenuiflora* is able to accumulate Na^+^, K^+^, and organic acids in vacuoles, as well as proline, betaine, and soluble sugar in the protoplasm to maintain osmotic homeostasis^4^,^24^. Importantly, some genes were found to be involved in ion compartmentalization in stressed *P. tenuiflora. PutCAX1* encoding a Ca^2+^/H^+^ antiporter in the vacuolar membrane was proposed to play a role in Ca^2+^, Ba^2+^, and Zn^2+^ transportation^26^. Besides, *PutNHX* encoding a vacuolar Na^+^/H^+^ antiporter was found to be responsible for Na^+^ sequestration into the vacuole^27^. Moreover, the vacuolar Na^+^/H^+^ antiporter might be involved in pH regulation under alkaline salt conditions due to higher NaHCO_3_-induced expression level of *PutNHX* in *P. tenuiflora* roots compared with that under NaCl condition^27^. To cope with the high pH of micro-environment around roots under alkali stress, *P. tenuiflora* can pump out H^+^ through the ATPase system on the cell membrane or secrete acidic metabolites such as organic acids^4^. Moreover, *P. tenuiflora* can also accumulate organic acids or other acidic metabolites in cells to adjust to the internal pH^4^. In addition, some antioxidant pathways were found to be activated in *P. tenuiflora* to scavenge excessive reactive oxygen species (ROS) caused by salt/alkali stress^24^,^25^,^28^. Two genes, *PutAPX* encoding ascorbate peroxidase (APX) and *PutMT2* encoding a type-2 metallothionein-like protein, which are all involved in ROS scavenging, were identified in *P. tenuiflora*. Over-expressing *PutAPX* and *PutMT2* in transgenic *Arabidopsis thaliana* and yeast increased the tolerance to H_2_O_2_, NaCl, and NaHCO_3_^29^,^30^.

Besides the aforementioned genes, more candidate genes and/or proteins involved in alkali tolerance have been found in *P. tenuiflora* using high-throughput transcriptomic and proteomic approaches^17^,^24^,^25^,^31^,^32^. The genes participated in ion transport, Fe acquisition, metabolism, and defense were up-regulated in seedlings under 20 mM NaHCO_3_^31^. Besides, the genes involved in metabolism, cell growth and photosynthesis were strongly affected in leaves stressed by 450 mM NaHCO_3_^32^. Similarly, Na_2_CO_3_-responsive genes involved in metabolism, signal transduction, transcription, and cell rescue and defense were overrepresented in seedlings^17^. The expression patterns of Na_2_CO_3_-responsive genes imply that dynamic regulation of photosynthesis, cytoskeleton, kinase-mediated signaling, and transcription, maintenance of redox and osmotic homeostasis, and the increased ability to regulate intracellular pH homeostasis and synthesize citric acid are important in *P. tenuiflora* to cope with alkali stress^17^. In addition, our previous comparative proteomic studies based on two-dimensional gel electrophoresis have identified 93 NaCl-responsive and 43 Na_2_CO_3_-responsive proteins in *P. tenuiflora* leaves, respectively. These results reveal that NaCl- and Na_2_CO_3_-responsive regulatory mechanisms in *P. tenuiflora* leaves share some common pathways, including declined photosynthesis, activation of antioxidant systems, ion exclusion and compartmentalization, as well as enhanced energy supply^24^,^25^. Despite the progress, the precise molecular mechanisms and regulatory networks of alkali tolerance in *P. tenuiflora* roots are still unknown.

In the present study, we investigated the Na_2_CO_3_-responsive characteristics in *P. tenuiflora* roots using physiological approaches combined with isobaric tags for relative and absolute quantification (iTRAQ)-based quantitative proteomic approach. By integrating the physiological and proteomic results, some unique Na_2_CO_3_-responsive pathways including alkali signal transduction, ROS scavenging, ionic and osmotic homeostasis, and protein synthesis and turnover were observed to play vital roles in Na_2_CO_3_ tolerance in *P. tenuiflora* roots. These results provide novel insights into the alkali tolerance mechanisms in *P. tenuiflora*, which is required for further studies on improving the crop alkali tolerance.

## Results

### Root growth and biomass of *P. tenuiflora* under Na_2_CO_3_

**Figure 1:**
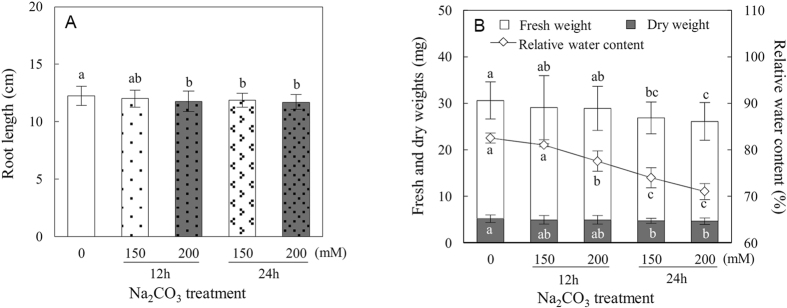
Root biomass of *Puccinellia tenuiflora* seedlings grown under Na_2_CO_3_ conditions. (**A**) Root length (n = 35) and (**B**) fresh weight (white columns) (n = 35), dry weight (gray columns) (n = 35), and relative water content (diamonds) (n = 3). The values were determined under control, 150 mM Na_2_CO_3_ for 12 h, 200 mM Na_2_CO_3_ for 12 h, 150 mM Na_2_CO_3_ for 24 h, and 200 mM Na_2_CO_3_ for 24 h. The values are presented as means ± standard deviation. Different letters indicate significant differences among different treatments (*p* < 0.05).

*P. tenuiflora* is a monocotyledonous halophyte with a high alkali tolerance. It can tolerate up to 150 mM Na_2_CO_3_ (pH 11.0) but not 200 mM or more Na_2_CO_3_ (pH 11.0) for 6 days^17^. In this study, 50-day-old seedlings were exposed to the tolerant level (150 mM) and lethal level (200 mM) of Na_2_CO_3_ for 12 h and 24 h, respectively. Root biomass was measured after Na_2_CO_3_ treatments. The root length was not significantly affected by 150 mM Na_2_CO_3_ for 12 h, but was decreased by 4%, 3.3% and 4.6% under 200 mM Na_2_CO_3_ for 12 h, 150 mM for 24 h, and 200 mM for 24 h, respectively (Fig. 1A). In addition, the fresh weight (Fw) and dry weight (Dw) of roots did not change significantly under Na_2_CO_3_ for 12 h (Fig. 1B). However, the root Fw was decreased by 12.9% and 15.6% under 150 mM and 200 mM Na_2_CO_3_ for 24 h, respectively. Similarly, the Dw of roots was also decreased by 9.1% and 10.6% under these two conditions (Fig. 1B). Besides, the relative water content (RWC) in roots was decreased by 6%, 10.3%, and 13.9% under 200 mM Na_2_CO_3_ for 12 h, 150 mM for 24 h, and 200 mM for 24 h, respectively (Fig. 1B). The data show that the root biomass and growth are apparently inhibited under Na_2_CO_3_ stress.

### Na^+^, K^+^, Ca^2+^, and Mg^2+^ contents in Na_2_CO_3_-stressed roots

**Figure 2:**
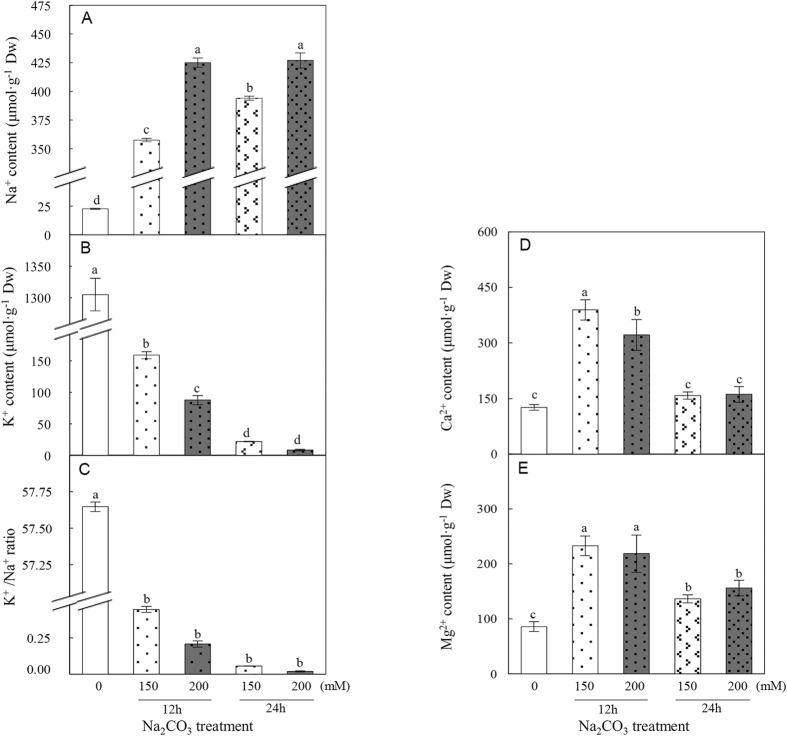
Effect of Na_2_CO_3_ on ion contents in *Puccinellia tenuiflora* roots. (**A**) Na^+^ content; (**B**) K^+^ content; (**C**) K^+^/Na^+^ ratio; (**D**) Ca^2+^ content; and (**E**) Mg^2+^ content. The values were determined under control, 150 mM Na_2_CO_3_ for 12 h, 200 mM Na_2_CO_3_ for 12 h, 150 mM Na_2_CO_3_ for 24 h, and 200 mM Na_2_CO_3_ for 24 h. The values are presented as means ± standard deviation (n = 4). Different letters indicate significant differences among different treatments (*p* < 0.05).

To monitor the ion homeostasis affected by Na_2_CO_3_ stress, the contents of Na^+^, K^+^, Ca^2+^, and Mg^2+^ in *P. tenuiflora* roots were measured. The Na^+^ content in Na_2_CO_3_-stressed roots was significantly increased by 15.8, 18.8, 17.4, and 18.9-fold under the four Na_2_CO_3_ conditions, respectively (Fig. 2A). This was accompanied by a decline in the root K^+^ content by about 8.2-, 14.9-, 59.0-, and 152.1-fold, respectively (Fig. 2B). The K^+^/Na^+^ ratio was also dramatically decreased under Na_2_CO_3_ stress (Fig. 2C). This implies that the uptake of K^+^ into roots is inhibited by the increasing Na^+^. In addition, the Ca^2+^ content was significantly increased after 12 h of Na_2_CO_3_ stress (Fig. 2D), and the Mg^2+^ content was increased under all of the four Na_2_CO_3_ conditions (Fig. 2E).

### Osmolyte contents in roots under Na_2_CO_3_

**Figure 3:**
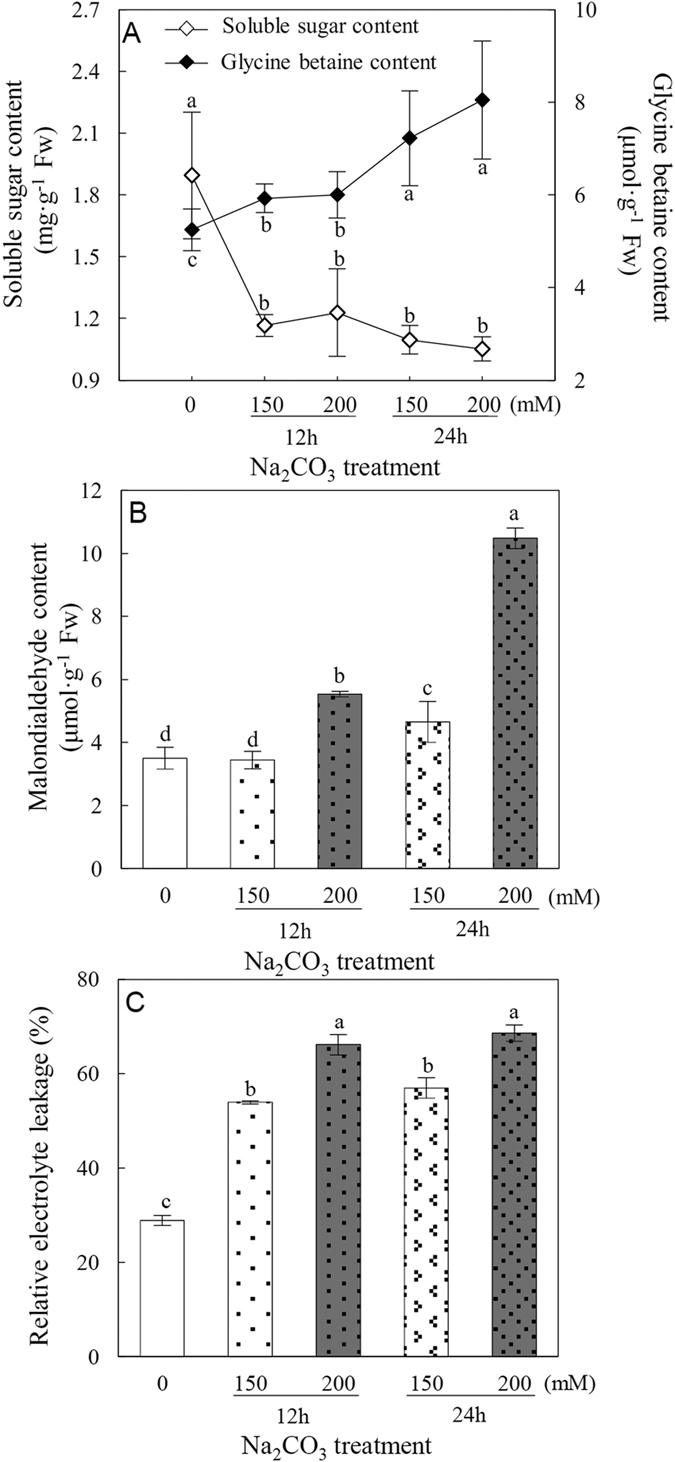
Effects of Na_2_CO_3_ on contents of (A) soluble sugar (white diamonds), glycine betaine (black diamonds), (B) malondialdehyde, and (C) relative electrolyte leakage in *Puccinellia tenuiflora* roots. The values were determined under control, 150 mM Na_2_CO_3_ for 12 h, 200 mM Na_2_CO_3_ for 12 h, 150 mM Na_2_CO_3_ for 24 h, and 200 mM Na_2_CO_3_ for 24 h. The values are presented as means ± standard deviation (n = 3). Different letters indicate significant differences among different treatments (*p* < 0.05).

The accumulation of osmolytes within plant cells is involved in alleviating alkali-induced osmotic stress. The contents of two important osmolytes, soluble sugar and glycine betaine, were measured in *P. tenuiflora* roots under Na_2_CO_3_ stress. The soluble sugar content was dramatically decreased in the Na_2_CO_3_-stressed roots (Fig. 3A). In contrast, the glycine betaine content was significantly increased in roots under Na_2_CO_3_ stress (Fig. 3A). This suggests that accumulating glycine betaine in *P. tenuiflora* roots may contribute to reestablish the osmotic balance under Na_2_CO_3_.

### Effect of Na_2_CO_3_ on root membrane integrity

To assess the impact of Na_2_CO_3_ stress on membrane integrity of *P. tenuiflora* roots, the malondialdehyde (MDA) content and relative electrolyte leakage (REL), two reliable indicators for alkali salt-induced membrane damage, were measured. Our data showed that MDA content did not change significantly after 150 mM Na_2_CO_3_ treatment for 12 h, but was significantly increased under 200 mM Na_2_CO_3_ for 12 h, 150 mM for 24 h, and 200 mM for 24 h (Fig. 3B). As to REL, a substantial increase was observed in *P. tenuiflora* roots under all the four Na_2_CO_3_ treatments (Fig. 3C). These results indicate that the membrane integrity of *P. tenuiflora* roots is damaged by Na_2_CO_3_ stress.

### Antioxidant enzyme activities in roots in response to Na_2_CO_3_

**Figure 4:**
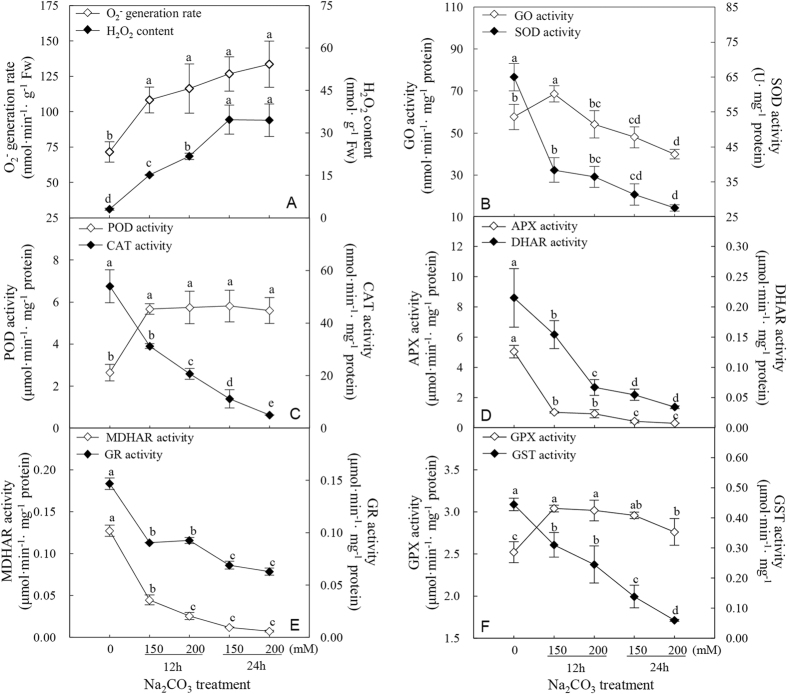
Effects of Na_2_CO_3_ on ROS production and antioxidant enzyme activities in *Puccinellia tenuiflora* roots. (**A**) O_2_^−^ generation rate (white diamonds) and H_2_O_2_ content (black diamonds); (**B**) glycolate oxidase (GO) (white diamonds) and superoxide dismutase (SOD) (black diamonds) activities; (**C**) peroxidase (POD) (white diamonds) and catalase (CAT) (black diamonds) activities; (**D**) ascorbate peroxidase (APX) (white diamonds) and dehydroascorbate reductase (DHAR) (black diamonds) activities; (**E**) monodehydroascorbate reductase (MDHAR) (white diamonds) and glutathione reductase (GR) (black diamonds) activities; and (**F**) glutathione peroxidase (GPX) (white diamonds) and glutathione S-transferase (GST) (black diamonds) activities. The values were determined under control, 150 mM Na_2_CO_3_ for 12 h, 200 mM Na_2_CO_3_ for 12 h, 150 mM Na_2_CO_3_ for 24 h, and 200 mM Na_2_CO_3_ for 24 h. The values are presented as means ± standard deviation (n = 3). Different letters indicate significant differences among different treatments (*p* < 0.05).

Since alkali salt imposes oxidative stress on plants by inducing the formation of ROS^33^, the O_2_^−^ generation rate and H_2_O_2_ content were measured in *P. tenuiflora* roots. The data showed that Na_2_CO_3_ caused dramatic increases in O_2_^−^ and H_2_O_2_ production in roots under all the four treatments (Fig. 4A), indicating that Na_2_CO_3_ resulted in oxidative damage to *P. tenuiflora* roots.

To determine the response of ROS scavenging system to oxidative stress induced by Na_2_CO_3_, the activities of various antioxidant enzymes including glycolate oxidase (GO), superoxide dismutase (SOD), peroxidase (POD), catalase (CAT), AsA-glutathione (GSH) cycle-related enzymes, glutathione peroxidase (GPX), and glutathione S-transferase (GST) were measured. The activity of GO was initially enhanced under 150 mM Na_2_CO_3_ for 12 h, but decreased after 24 h of Na_2_CO_3_ treatment (Fig. 4B). A substantial decrease in SOD activity was observed under Na_2_CO_3_ treatment (Fig. 4B). In contrast, POD activity displayed a significant increase in Na_2_CO_3_-stressed roots, while CAT activity was decreased after Na_2_CO_3_ treatment (Fig. 4C). In addition, the activities of APX, monodehydroascorbate reductase (MDHAR), dehydroascorbate reductase (DHAR), and glutathione reductase (GR) involved in AsA-GSH cycle all showed dramatic decreases in roots (Fig. 4D,E). The GPX activity was significantly increased, but GST activity was severely inhibited in roots under Na_2_CO_3_ stress (Fig. 4F). These results indicate that POD and GPX pathways have been initiated, while GO, SOD, CAT, AsA-GSH cycle, and GST pathways are inhibited in *P. tenuiflora* roots in response to Na_2_CO_3_ stress.

### Identification of Na_2_CO_3_-responsive proteins using iTRAQ -based liquid chromatography-tandem mass spectrometry (LC-MS/MS)

**Figure 5:**
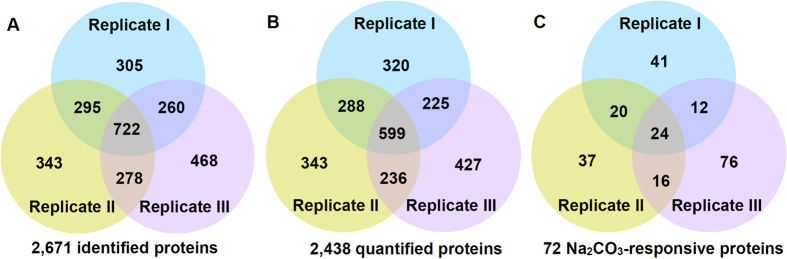
Venn diagram analysis of protein identification and quantification in three biological replicates. (**A**) The number of identified proteins with at least 95% confidence in three independent biological replicates. (**B**) The number of quantified proteins with at least 95% confidence in three independent biological replicates. (**C**) The number of Na_2_CO_3_-responsive proteins in three independent biological replicates.

To explore the proteomic changes in *P. tenuiflora* roots in response to Na_2_CO_3_ stress, we employed the iTRAQ-based proteomics approach (Supplementary Fig. S1). Protein abundance profiles in roots under control, 150 mM and 200 mM Na_2_CO_3_ treated for 12 h and 24 h were analyzed in three independent biological replicates. The ProteinPilot cut-off score for proteins identified was set at 1.3, which corresponded to a confidence level of 95%. The proteins with similar protein family name and/or amino acid sequence but identified from only one replicate were taken as one unique protein to avoid the redundancy from combination of replicates. Finally, 2,671 non-redundant proteins were included in the dataset of *P. tenuiflora* roots (Fig. 5A; Supplementary Table S1). In this dataset, a total of 1,594 function unknown proteins were function annotated by searching against the NCBI non-redundant protein database using PSI and PHI-BLAST programs, and then all the 2,671 non-redundant proteins were classified into 17 functional categories based on BLAST alignment, information searching from KEGG pathway database, UniProt database, Gene Ontology, as well as literature (Supplementary Table S1).

Of the proteins identified, a total of 2,438 proteins were quantified in at least one of the replicates, and 1,348 proteins were quantified in at least two of the three independent replicates (Fig. 5B; Supplementary Table S1). A total of 226 Na_2_CO_3_-responsive proteins were identified and quantified in at least one of the three independent replicates under Na_2_CO_3_ stress based on the ratio fold change ≥1.5 and *p* < 0.05. Among them, 72 Na_2_CO_3_-responsive proteins were reproducibly identified in at least two replicates (Fig. 5C; Table 1 and Supplementary Table S2).

### Na_2_CO_3_-responsive signaling and metabolic processes revealed from protein patterns

Broadly, 72 Na_2_CO_3_-responsive proteins covered a wide range of molecular functions, including signaling, ROS scavenging, transportation, chromosome assembly, transcription, protein synthesis, protein processing, protein degradation, carbohydrate and energy metabolism, amino acid metabolism, fatty acid metabolism, and other metabolisms (Table 1 and Supplementary Table S2). Among them, protein synthesis-related proteins accounted for the largest group (26% of Na_2_CO_3_-responsive proteins) (Table 1). The abundance change patterns of Na_2_CO_3_-responsive proteins show that multiple signaling and metabolic pathways are modulated in roots to cope with stress.

Signaling transduction and vesicle trafficking are affected by Na_2_CO_3_ stress. Six Na_2_CO_3_-responsive proteins involved in signal transduction were identified, including four calcium binding proteins, protein kinase and phosphatase. Among them, two calcium binding proteins, developmentally regulated plasma membrane polypeptide (DREPP) and calreticulin (CRT)-like protein were increased, but the other two proteins (CRT and calmodulin) were decreased under Na_2_CO_3_ stress (Table 1). Calcium-dependent protein kinase (CDPK) and serine/threonine-protein phosphatase (STPP) involved in protein phosphorylation and dephosphorylation were increased in Na_2_CO_3_-stressed roots. In addition to calcium signaling, ROS also work as signal molecules for alkali-responsive regulation. The increases of two ROS scavenging-related proteins, 2-Cys peroxiredoxin (PrxR) and GR, were detected under Na_2_CO_3_ stress (Table 1). Both PrxR and GR are responsible for H_2_O_2_ reduction, which is supposed to help maintain cellular ROS homeostasis. In addition, four Na_2_CO_3_-responsive proteins involved in cellular transportation were decreased in roots under stress (Table 1). They were PM H^+^-ATPase (P-ATPase) involved in proton transport, vesicle-associated membrane protein (VAMP) involved in membrane fusion, dynamin-related protein (DRP) associated with membrane trafficking, and mitochondrial phosphate transporter (MPT) in charge of Pi uptake. However, Rab1 related to cellular trafficking was increased in Na_2_CO_3_-stressed roots (Table 1).

Under the stress, gene expression and protein synthesis are altered in roots. We found four proteins involved in chromosome assembly and transcription were affected by Na_2_CO_3_ stress (Table 1). Among them, Na_2_CO_3_-decreased histone H2B and Na_2_CO_3_-increased nucleosome assembly protein (NAP) were involved in chromosome assembly. In addition, Na_2_CO_3_-increased transcription factor purine-rich alpha 1 (PURα1) and RNA recognition motif (RRM) could contribute to enhance specific gene transcription under stress condition. Importantly, we also identified thirty proteins involved in protein synthesis and turnover in Na_2_CO_3_-treated roots (Table 1). Among them, seventeen out of nineteen proteins related to protein synthesis were ribosomal protein subunits. They were all decreased in roots under Na_2_CO_3_ stress (Table 1). However, eukaryotic initiation factor 2b (eIF2b) involved in the initiation phase of eukaryotic translation was increased under Na_2_CO_3_ stress, and eIF3 was increased by Na_2_CO_3_ treatment for 24 h when compared with 12 h. In addition, four protein folding and processing-related proteins, nascent polypeptide-associated complex (NAC), chaperonin 60 (CPN60), T-complex protein 1 (TCP1), and Hsp90 were all decreased after Na_2_CO_3_ treatments. Furthermore, seven alkali-responsive proteins involved in protein degradation were also identified (Table 1). Among them, 26S protease regulatory subunit 4 (P26S4) and 6B (P26S6B), and 26S protease regulatory subunit-like protein (P26SLP) were decreased, but 26S proteasome regulatory subunit S2 (P26S2) and proteasome subunit alpha type (PSA), as well as ATPase family associated with various cellular activities (AAA+ ATPase) were increased in roots under Na_2_CO_3_. Additionally, methionine aminopeptidase (MAP) was decreased in roots treated with 150 mM and 200 mM Na_2_CO_3_ for 12 h when compared with 150 mM Na_2_CO_3_ for 24 h. This implies that protein synthesis, processing, and turnover are generally inhibited under Na_2_CO_3_.

Seven Na_2_CO_3_-responsive proteins involved in carbohydrate and energy metabolism were detected (Table 1). Among them, phosphoglycerate kinase was decreased under 150 mM Na_2_CO_3_ for 24 h when compared with 200 mM Na_2_CO_3_ for 12 h. In addition, pyruvate kinase (accession number F2CS51) was increased under 200 mM Na_2_CO_3_ for 24 h when compared with 150 mM Na_2_CO_3_ for 12 h. However, another pyruvate kinase (accession number F2CX32) was increased in Na_2_CO_3_-stressed roots. Besides, dihydrolipoyllysine-residue succinyltransferase component of 2-oxoglutarate dehydrogenase complex in the tricarboxylic acid (TCA) cycle, transaldolase in the pentose phosphate pathway, and sorbitol dehydrogenase associated with sugar metabolism were increased under Na_2_CO_3_ stress. While, ATP synthase gamma chain was decreased under Na_2_CO_3_ stress.

We identified five Na_2_CO_3_-responsive proteins involved in amino acid metabolism (Table 1). Chorismate synthase, glutamine synthetase, and aspartate aminotransferase were decreased, but *O*-acetylserine (thiol) lyase of cysteine synthase complex and pyrroline-5-carboxylate reductase were increased. Moreover, three fatty acid metabolism-related proteins were decreased, including 3-ketoacyl-CoA thiolase-like protein, ATP citrate lyase, and leukotriene A4 hydrolase. Additionally, we also found ten proteins involved in other metabolisms, most of which were increased in *P. tenuiflora* roots under Na_2_CO_3_ stress (Table 1).

### Protein-protein interaction (PPI) network

**Figure 6:**
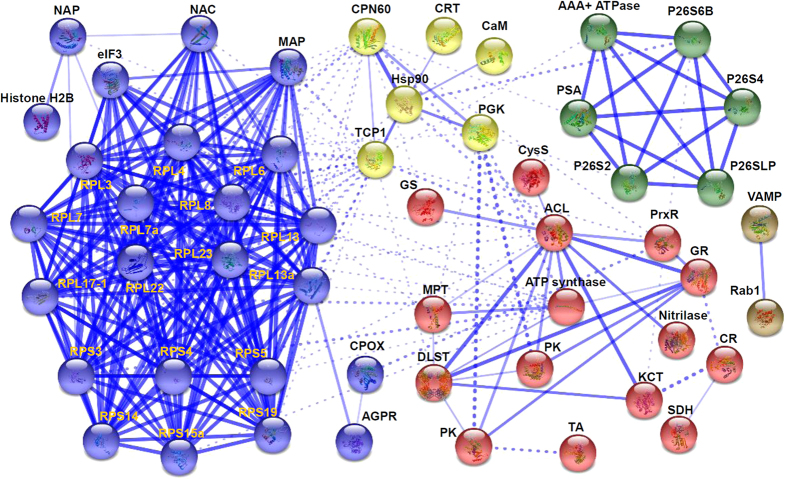
Visualization of protein-protein interaction (PPI) network of differentially abundant proteins in *Puccinellia tenuiflora* roots using STRING analysis (confidence mode). A total of 53 differentially abundant proteins represented by homologous proteins from *Arabidopsis* are shown in PPI network. The nodes represent proteins, and different protein groups are indicated in different colors. The lines represent the predicted functional associations. Strong associations are represented by thicker lines. Detailed information on protein names and abbreviations can be found in Table 1.

PPI network for Na_2_CO_3_-responsive proteins was visualized using STRING analysis based on homologous proteins in *Arabidopsis* (Fig. 6; Supplementary Table S2). Out of the 72 proteins, 53 proteins were depicted in the STRING database based on published literature, genome analysis of domain fusion, phylogenetic profiling/homology, gene neighborhood, co-occurrence, co-expression, and other experimental evidence. In the protein networks, stronger associations are represented by thicker lines (Fig. 6). Four main interactive clusters were formed among these proteins (Fig. 6). In Model 1 (yellow nodes), proteins involved in calcium signaling (CRT and calmodulin), protein processing (CPN60, TCP1, and Hsp90), and glycolysis (phosphoglycerate kinase) appeared close links (Fig. 6). This indicates that active protein turnover is crucial for signal transduction in Na_2_CO_3_-stressed roots. Model II (red nodes) included fifteen proteins belonging to ROS scavenging (PrxR and GR), transportation (MPT), carbohydrate metabolism (pyruvate kinase, dihydrolipoyllysine-residue succinyltransferase, transaldolase, sorbitol dehydrogenase, and ATP synthase), amino acid metabolism (glutamine synthetase and *O*-acetylserine (thiol) lyase of cysteine synthase complex). Additionally, four proteins participating in other metabolisms were also assigned in Model 2 (Fig. 6). Moreover, chromosome assembly proteins (histone H2B and NAP) and the members of protein synthesis machine (eIF3 and ribosomal proteins), as well as ribosome-associated chaperone NAC and aminopeptidase MAP were closely linked in Model III (blue nodes) (Fig. 6). In addition, five subunits of 26S proteasome (P26S2, P26S4, P26S6B, P26SLP, and PSA) and AAA+ ATPase were assigned in Model IV (green nodes). These indicate that protein synthesis and turnover play important roles in roots under alkali stress. Besides, interaction between two transportation-related proteins, VAMP and Rab1, was also predicted in the network.

### Homologous gene expression of Na_2_CO_3_-responsive proteins

**Figure 7:**
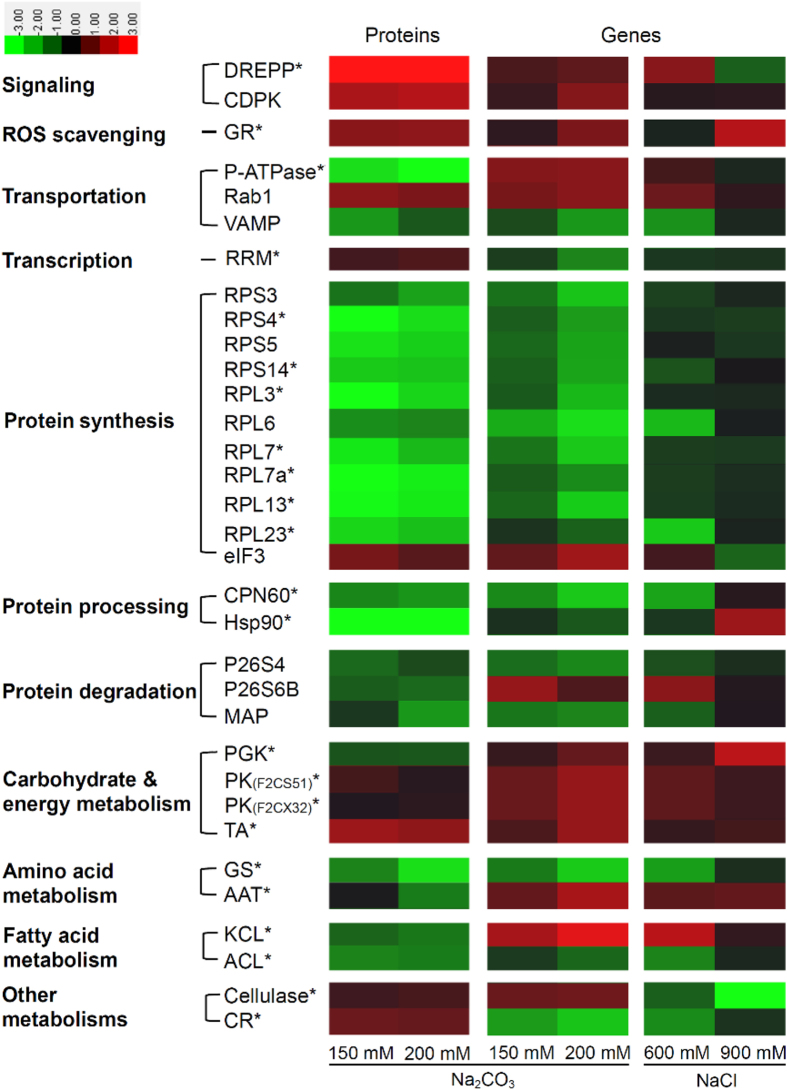
Expression pattern of 33 Na_2_CO_3_-responsive proteins and their corresponding genes under alkaline and salt stresses. The columns represent different treatment conditions. They were 150 mM and 200 mM Na_2_CO_3_ for 12 h, as well as 600 mM and 900 mM NaCl for 12 h. The rows represent individual proteins and corresponding genes. Abbreviations of protein names and metabolic pathways are listed on the left side. The scale bar indicates log2 transformed relative expression levels of proteins and genes. The increased and decreased abundances of proteins and genes are represented in red and green, respectively. The color intensity increases with increasing abundant differences. Protein name marked with an asterisk represents the protein has homologous gene in cDNA dataset of *Puccinellia tenuiflora*. Accession numbers of two isoforms of pyruvate kinase were indicated in the brackets. Please see Table 1 for protein name abbreviations. Detailed information can be found in Supplementary Table S3.

After sequence alignment analysis using TBLASTN algorithm, 69 homologous genes of Na_2_CO_3_-responsive proteins were found in the cDNA library of *P. tenuiflora* treated with 100 mM Na_2_CO_3_. Among them, 23 homologous genes were found to be differentially expressed at more than two-fold in seedlings under NaCl (600 mM and 900 mM for 12 h) and Na_2_CO_3_ (150 mM and 200 mM for 12 h) treatments based on microarray analysis (Supplementary Table S3). In addition, another ten differentially expressed genes in the microarray results were supposed to have similar function with the encoding genes of Na_2_CO_3_-responsive proteins based on protein functional domain analysis (Supplementary Table S3). Altogether, the correlation between 33 Na_2_CO_3_-responsive proteins and their corresponding genes were evaluated based on the comparison of proteomic and microarray results (Fig. 7). The results showed that nine proteins appeared in the increasing trends consistent with their corresponding genes, including two signal transduction-related proteins (DREPP and CDPK), a ROS scavenging enzyme (GR), a transportation-related Rab1, an eIF3 for protein synthesis, three carbohydrate/energy metabolic enzymes (two pyruvate kinases and a transaldolase), and a cell wall dynamics-related cellulase (Fig. 7). Furthermore, 17 Na_2_CO_3_-decreased proteins have similar trends as the corresponding genes, such as VAMP, ten ribosome proteins, two protein processing-related CPN60 and Hsp90, two enzymes for protein degradation (P26S4 and MAP), a glutamine synthetase for amino acid metabolism, as well as an ATP citrate lyase for fatty acid metabolism (Fig. 7). However, seven proteins showed opposite expression trends with their corresponding genes. Among them, five alkali-decreased proteins (i.e., P-ATPase, P26S6B, phosphoglycerate kinase, aspartate aminotransferase, and 3-ketoacyl-CoA thiolase-like protein) appeared induced at the transcriptional level, and the gene expression of two alkali-increased proteins (RRM and carbonyl reductase) were reduced. Interestingly, most of these genes, except for the cellulase and eIF3, showed similar expression trends in response to various Na_2_CO_3_ and NaCl treatments (Fig. 7). All these results indicate that the levels of most Na_2_CO_3_-responsive proteins were consistent with the corresponding gene expression levels in *P. tenuiflora* seedlings.

## Discussion

### Alkali salt is more likely to cause serious stress than neutral salt

The activity of root system under stress conditions is critical for the plant survival and optimal growth^34^. *P. tenuiflora* is a monocotyledonous halophyte with high tolerance to alkaline stress. *P. tenuiflora* plants grow well under 50 mM Na_2_CO_3_ (pH 11.0) and could tolerate up to 150 mM Na_2_CO_3_ (pH 11.0) for 6 days^17^. The relative growth rate of *P. tenuiflora* roots was increased under 60 mM mixed alkali salt stress (NaHCO_3_ and Na_2_CO_3_) for 7 days, but it was decreased at higher concentrations (120–240 mM)^4^. Consistently, we found that the root growth was slightly inhibited under 150 mM Na_2_CO_3_ for 24 h and 200 mM Na_2_CO_3_ for 12 h and 24 h (Fig. 1A,B). These results suggest that the root growth of *P. tenuiflora* might be promoted under alkali stress with as high as 60 mM Na^+^, but inhibited at higher alkali concentrations. Different from halophyte *P. tenuiflora*, root growth of glycophyte sunflower was reduced under 5–15 mM Na_2_CO_3_ stress^7^. This implies that halophyte roots probably exhibit higher alkali tolerance than glycophyte.

In addition to alkali stress, *P. tenuiflora* can tolerate up to 600 mM NaCl for 6 days, but 900 mM NaCl would lead to the death of *P. tenuiflora*^17^. The relative growth rate of *P. tenuiflora* root was increased after 60 mM and 120 mM mixed neutral salt stress (NaCl and Na_2_SO_4_)^4^. However, the root biomass of *P. tenuiflora* was decreased after treated with 150 and 200 mM NaCl for 7 days^2^, and the relative growth rate of roots was significantly inhibited after 240 mM mixed salt stress (NaCl and Na_2_SO_4_)^4^. These results indicate that *P. tenuiflora* can tolerate higher level of neutral salts when compared with the level of alkali salts. This is probably because alkali stress causes an additional high pH stress to plants in addition to ionic and osmotic stresses^35^. In order to survive from such a severe stress, the activation and cooperation of multiple salt-resistant pathways are required to support optimal growth under alkali stress.

### Ca^2+^ signaling and reversible protein phosphorylation are crucial for Na_2_CO_3_ response in roots

**Figure 8:**
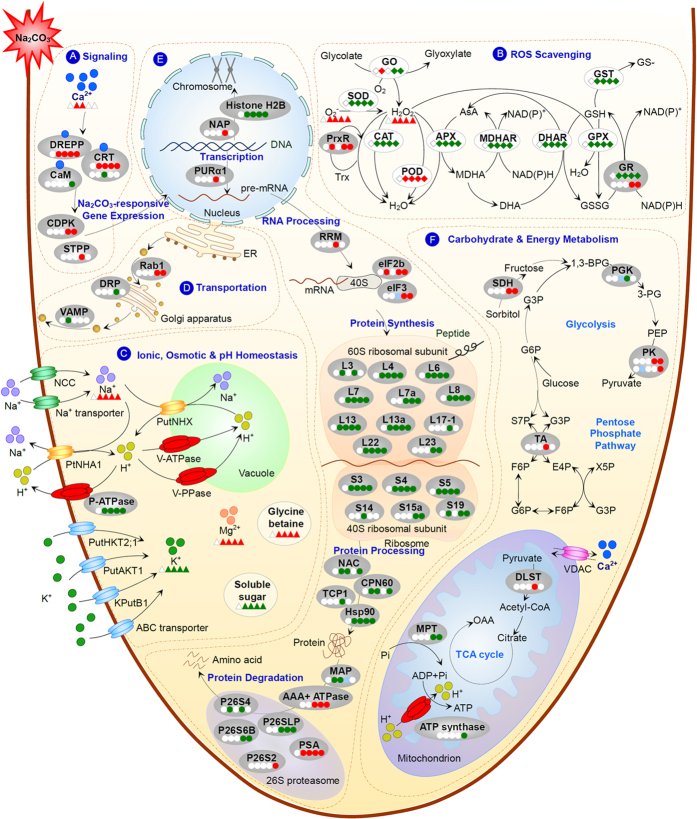
Na_2_CO_3_-responsive mechanism in roots of *Puccinellia tenuiflora* revealed by iTRAQ-based proteomics. The solid line indicates single-step reaction, and the dashed line indicates multi-step reactions. Relative protein abundances, enzyme activities, and substrate contents in corresponding treatments compared with control are marked with circles, diamonds, and triangles in white (unchanged), red (increased), and green (decreased), respectively. Most of the protein abundance changes were compared with control condition (the left white circle), but the abundance changes of eIF3, PGK, PK, and MAP were compared with other treatment conditions which were marked with blue circles. Five circles/diamonds/triangles from left to right represent different treatment conditions including control, 150 mM Na_2_CO_3_ for 12 h, 200 mM Na_2_CO_3_ for 12 h, 150 mM Na_2_CO_3_ for 24 h, and 200 mM Na_2_CO_3_ for 24 h, respectively. (**A**) signaling; (**B**) ROS scavenging; (**C**) ionic, osmotic, and pH homeostasis; (**D**) transportation; (**E**) protein synthesis and turnover; (**F**) carbohydrate and energy metabolism. Abbreviations: 1,3-BPG, 1,3-bisphosphoglycerate; 3-PG, 3-phosphoglycerate; ABC transporter, ATP-binding cassette transporter; AKT, *Arabidopsis* K^+^ transporter; APX, ascorbate peroxidase; AsA, ascorbate; CAT, catalase; DHA, dehydroascorbate; DHAR, dehydroascorbate reductase; E4P, erythrose 4-phosphate; ER, endoplasmic reticulum; F6P, fructose 6-phosphate; G3P, glyceraldehyde 3-phosphate; G6P, glucose 6-phosphate; GO, glycolate oxidase; GPX, glutathione peroxidase; GSH, glutathione; GSSG, oxidized glutathione; GST, glutathione S-transferase; HKT, high-affinity K^+^ transporter; KPutB, K^+^ channel β subunit from *Puccinellia tenuiflora*; MDHA, monodehydroascorbate; MDHAR, monodehydroascorbate reductase; NCC, nonselective cation channel; NHA, Na^+^/H^+^ antiporter; NHX, Na^+^/H^+^ exchanger; OAA, oxaloacetic acid; PEP, phosphoenolpyruvate; POD, peroxidase; S7P, sedoheptulose 7-phosphate; SOD, superoxide dismutase; TCA, tricarboxylic acid; Trx, thioredoxin; V-ATPase, vacuolar-type H^+^-transporting ATPase; VDAC, voltage-dependent anion channel protein; V-PPase, vacuolar proton-inorganic pyrophosphatase; X5P, xylulose 5-phosphate. Please see Table 1 for abbreviations of proteins identified in this study.

In the complicated salt-responsive signaling networks, the alteration of intracellular Ca^2+^ levels, the Ca^2+^ interaction with calcium-binding proteins, and the activation of Ca^2+^-regulated protein phosphorylation cascades are all vital for modulating specific salt-responsive gene expression^36^. Our data here provide important information for underlying Na_2_CO_3_-responsive Ca^2+^ signaling pathways in *P. tenuiflora* roots (Fig. 8A).

In general, transient increase in cytosolic Ca^2+^ is considered to be an early response to Na^+^ increase in roots^8^. We found that Ca^2+^ content was increased in *P. tenuiflora* roots under 150 mM and 200 mM Na_2_CO_3_ for 12 h, but it was unchanged after 24 h treatment (Fig. 2D). Interestingly, in roots of halophyte *Kosteletzkya virginica*, Ca^2+^ level also did not change under 100 mM NaCl for 26 days^37^. This implies that the variation of Ca^2+^ level in halophyte roots transmits an important transient signal to trigger certain alkali-/salt- responsive gene expression.

Consistent with the transient increase of Ca^2+^ level, our proteomic results revealed that two calcium-binding proteins, DREPP and CRT-like protein, which involved in receiving Ca^2+^ signal, were increased under various Na_2_CO_3_ stress conditions (Table 1). Besides, a homologous gene of DREPP was induced in *P. tenuiflora* in response to alkali and salt stresses^17^ (Fig. 7). Similarly, DREPP was also increased in rice roots under NaCl stress^38^. This might facilitate the transduction of calcium signal for initiating downstream alkali-/salt- responsive gene expression^39^. However, *DREPP* gene expression was decreased in wild soybean roots under 50 mM NaHCO_3_^12^, indicating the different alkali-responsive patterns of DREPP at the gene and protein levels. Besides, we found CRT was decreased under 200 mM Na_2_CO_3_ for 12 h (Table 1). Previous transcriptomic investigations have reported the diverse expression patterns of *CRT* genes in response to alkali stress. For example, the majority of *CRT* genes were induced in wild soybean roots under 50 mM NaHCO_3_^12^. However, 50 mM Na_2_CO_3_ treatment for 5 h led to the down-regulation of a *CRT* gene in maize roots^11^. All these results suggest that CRTs in roots are sensitive to alkali stresses, being regulated at both gene and protein levels in different plant species. Moreover, calmodulin, another Ca^2+^ signal transducer, was decreased in *P. tenuiflora* roots under Na_2_CO_3_ stress (Table 1). The down-regulation of *calmodulin* genes was also found in roots of halophyte *Limonium bicolor*^40^ and wild soybean^12^ under NaHCO_3_ stress. Moreover, the NaCl-decreased calmodulin was detected in roots of rice^38^, maize^41^, and tomato (*Solanum lycopersicum*)^42^. This suggests that calmodulin is a common member in salt- and alkali-responsive signaling pathways in roots.

It has been proposed that reversible protein phosphorylation cascades play important roles in Ca^2+^ signaling under salt stress^36^. CDPK, which can be activated directly by the binding of Ca^2+^ to its calmodulin-like domain^43^, is considered as one of the major conserved players in coupling inorganic Ca^2+^ signal to specific protein phosphorylation cascade^36^. In our results, CDPK was increased by Na_2_CO_3_ in *P. tenuiflora* roots (Table 1). Consistently, a *CDPK* gene was also induced in *P. tenuiflora* seedlings under Na_2_CO_3_^17^ (Fig. 7). A large amount of gain-of-function and loss-of-function studies have proved that various *CDPK* genes, such as *OsCDPK7*^44^, *OsCPK12*^45^, *OsCPK21*^46^, *AtCPK6*^47^ and *ZoCDPK1*^48^, were all positive regulators involved in plant salt tolerance. Among them, the *OsCDPK7*^44^ and *AtCPK6*^47^ are considered as the homologous genes of *PtCDPK* (Contig720)^17^, because their encoded proteins showed 61% and 62.1% identities, respectively, on the basis of amino acid sequence analysis (Supplementary Fig. S2). It has been proved that over-expressing *OsCDPK7*^44^ and *AtCPK6*^47^ enhanced the rice and Arabidopsis tolerance to salt stress. Thus, the Na_2_CO_3_-induced PtCDPK at protein and gene levels would function in enhancement of alkali tolerance. Similarly, a *CDPK* gene was up-regulated in roots of halophyte *L. bicolor* under 400 mM NaHCO_3_ for 48 h^40^. Three out of four *CDPK* genes identified in halophyte *T. hispida* roots were also up-regulated under 300 mM NaHCO_3_ for 12 h and 48 h^13^. However, in wild soybean roots, only about half of *CDPK* genes were up-regulated under 50 mM NaHCO_3_^12^, which might be due to its less alkali tolerance when compared with the halophytes mentioned above. Besides, STPP containing protein phosphatase type 1 (PP1) and keltch like domains, was also increased in *P. tenuiflora* roots under Na_2_CO_3_ treatment (Table 1). Interestingly, a *PP1 isoform 2* gene was increased while *PP1* genes were decreased under 300 mM NaHCO_3_ for 12 h and 24 h in roots of woody halophyte *T. hispida*^13^. In addition, the majority of *STPP* genes in wild soybean roots were decreased under 50 mM NaHCO_3_^12^. The diverse patterns of STPP in roots under various alkali stresses suggest that the rapid switch between protein phosphorylation and dephosphorylation happens transiently for modulating corresponding gene expression in the roots to cope with stress.

### Specific ROS scavenging pathways are employed in roots under Na_2_CO_3_

In salinity-stressed roots, the ROS level is dramatically elevated in cytosol, mitochondrion, peroxisome, and apoplast^33^. The accumulated ROS play a dual role in salt response, as toxic molecules causing oxidative damage and signaling molecules in the regulation of stress-responsive gene expression. Thus, the balance between ROS production and ROS scavenging is crucial to root growth under stress condition. In roots, ROS is mainly produced from over-reduction of the electron transduction chain in mitochondrion, while ROS detoxification depends on various ROS scavenging enzymes and antioxidants (e.g., AsA and GSH)^33^,^49^.

In this study, we found that oxidative stress triggered by Na_2_CO_3_ disrupted cellular membrane system and normal metabolism in roots. The O_2_^−^ and H_2_O_2_ levels were increased dramatically with the increase of Na_2_CO_3_ levels (Fig. 4A). The cell membrane appeared to be damaged by ROS, as evidenced from the increased root MDA content and REL (Fig. 3B,C). All these indicate that *P. tenuiflora* roots undergo serious oxidative stress when subjected to 150 mM and 200 mM Na_2_CO_3_. Importantly, among ten important enzymes in ROS scavenging system, only the activities of POD and GPX were increased, but those of SOD, CAT, APX, MDHAR, DHAR, GR, and GST were all decreased with the increasing Na_2_CO_3_ stress (Fig. 4B–F). This implies that most ROS scavenging pathways are inhibited under Na_2_CO_3_ stress (Fig. 8B). Thus, in these cases, the accumulated H_2_O_2_ could not be efficiently scavenged through the CAT pathway and AsA-GSH cycle, which were catalyzed by APX, MDHAR, DHAR, and GR (Fig. 8B). Considering the increased level of PrxR revealed from our proteomic results (Table 1), the extra H_2_O_2_ might be eliminated mainly through the POD, PrxR, and GPX pathways to cope with Na_2_CO_3_ stress. In addition, the decreased activity of GST was speculated to accelerate GSH accumulation for active GPX pathway in the roots (Fig. 8B). Similarly, the pathways of POD, PrxR, and GPX have been reported to be alkali-increased in roots of other plant species. For example, POD abundance was increased in tomato roots under 50 mM NaHCO_3_^42^, and several genes encoding PODs were induced in roots of woody halophyte *T. hispida*^13^ and wild soybean^12^ under NaHCO_3_. In addition, *PrxRs* were up-regulated in wild soybean roots under 50 mM NaHCO_3_^12^. All these indicate that the enhancement of POD, PrxR, and GPX pathways would facilitate the scavenging of ROS in alkali-stressed roots.

The Na_2_CO_3_-inhibited pathways found in this study, such as SOD pathway, CAT pathway, and AsA-GSH cycle, were also known to be alkali-/salt-inhibited in roots of other plants. For example, the NaHCO_3_-reduced genes of *CAT, APX, MDHAR*, and *GR* were reported in roots of *T. hispida*^13^ and wild soybean^12^. Generally, the protein abundances and/or activities of oxidative enzymes were inhibited under higher concentration or longer duration of stress. For example, the activity of GO was increased in *P. tenuiflora* roots under 150 mM Na_2_CO_3_ for 12 h, but decreased under 24 h treatment (Fig. 4B). GO acts as a H_2_O_2_ generator through catalyzing the oxidation of glycolate to glyoxylate. Our results indicate that the oxidation of glycolate is initially induced under 150 mM Na_2_CO_3_ for 12 h, but it is inhibited under severe Na_2_CO_3_ stress, leading to the accumulation of glycolate and reduction of H_2_O_2_ production (Fig. 8B). Similarly, under NaHCO_3_ stress, most *GSTs* in wild soybean roots were up-regulated at 3–6 h, but down-regulated after 12 h^12^.

Interestingly, our results revealed that the abundance of GR was increased (Table 1), which is consistent with the induced homologous *GR* gene in *P. tenuiflora*^17^ (Fig. 7), but its activity was decreased in *P. tenuiflora* roots under Na_2_CO_3_ stress (Figs 4E and 8B). This implies that the dynamic of ROS scavenging system and redox status in roots are transient, compartmental, and complicated in coping with the alkali stress. Studies of protein abundance, enzyme activities, and protein redox modulation at organelle level may facilitate a deep understanding of ROS scavenging and redox regulation.

### Modulation of ionic, osmotic, and pH homeostasis in roots under Na_2_CO_3_

Under alkali stress conditions, extra Na^+^ enters roots through nonselective cation channels and Na^+^ transporters, but the uptake of K^+^ is inhibited simultaneously^8^,^50^ (Fig. 8C). Especially, high pH has an additional influence on ionic and osmotic balance in roots^35^. Halophytes have developed diverse mechanisms to maintain intracellular ion homeostasis in roots, including maintaining K^+^ uptake, limiting Na^+^ entry, and enhancing Na^+^ exclusion and compartmentalization^35^. In *P. tenuiflora* roots, three genes encoding K^+^ transporters/channels have been characterized, including *PutHKT2;1*, *PutAKT1*, and *KPutB1* (Fig. 8C). Among them, *PutHKT2;1* encoding a PM-localized high-affinity K^+^ transporter was expressed mainly in roots, mediating a substantial K^+^ uptake under low external K^+^ concentration and in the presence of elevated Na^+ ^20. Moreover, the PM-localized *PutAKT1* encoding a hyperpolarization-activated K^+^-selective inward-rectifying channel, was also predominantly expressed in roots under both normal condition and NaCl stress^22^. The function of *PutAKT1* in salt tolerance was demonstrated from the enhanced cellular K^+^ uptake and reduced Na^+^ accumulation in the *PutAKT1* over-expressed *Arabidopsis* seedlings under salt stress^22^. In addition, *KPutB1* encoding a K^+^ channel β subunit was preferentially expressed in roots, and can be induced under 300 mM NaCl for 6–24 h^51^. *Arabidopsis* plants over-expressing *KPutB1* showed lower Na^+^ content and higher K^+^/Na^+^ ratio than that in the control plants under 75 mM NaCl^51^. Importantly, KPutB1 can interact with PutAKT1 and the yeast co-expressing *PutAKT1* and *KPutB1* showed better growth and higher K^+^ uptake ability than yeast expressing *PutAKT1* alone^51^. Besides, several Na_2_CO_3_-responsive ATP-binding cassette (ABC) transporter genes revealed from transcriptomic analysis may play a role in K^+^ transportation in *P. tenuiflora* seedlings^17^ (Fig. 8C). Furthermore, previous proteomic studies revealed several K^+^ transporters, such as voltage-gated potassium channel^52^, cyclic nucleotide-gated channel^53^, and ABC transporters^52^,^53^, were increased in roots of NaCl-stressed wheat (*Triticum aestivum*). All these highlight that enhancement of K^+^ uptake is a vital strategy in modulating ion homeostasis in roots to cope with salt and alkaline stresses. However, we found 150 mM and 200 mM Na_2_CO_3_ stress resulted in dramatic Na^+^ accumulation, K^+^ decline, and K^+^/Na^+^ ratio decrease in *P. tenuiflora* roots (Fig. 2A–C). This is different with our previous findings that the K^+^ content was increased in *P. tenuiflora* seedlings under 95 mM Na_2_CO_3_ for 7 days^25^. It needs to be further investigated whether it is the K^+^ uptake into roots were inhibited or the K^+^ in roots were rapidly transported to leave under higher concentration of Na_2_CO_3_.

In addition to the irreplaceable K^+^ required for diverse enzymatic processes, we found that the uptake of Ca^2+^ and Mg^2+^ was not inhibited in *P. tenuiflora* roots (Fig. 2D,E) and seedlings^25^ under Na_2_CO_3_. The increased Ca^2+^ in *P. tenuiflora* roots and seedlings under Na_2_CO_3_ and NaCl stresses^24^,^25^ might facilitate cell wall rigidity and PM integrity apart from its secondary messenger role^54^. Ca^2+^ transporters play a key role in regulating cellular Ca^2+^ levels to cope with salt and alkali stresses. An important Ca^2+^ transporter, voltage-dependent anion channel protein (VDAC) located in the mitochondrial outer membrane was reported to be affected by Na_2_CO_3_ and NaCl stresses (Fig. 8F). For instance, the expression of two genes encoding VDACs were inhibited in *P. tenuiflora* seedlings under Na_2_CO_3_ stress^17^. However, proteomic studies have revealed that VDACs were increased in NaCl-stressed roots of maize^41^ and wild tomato (*Solanum chilense*)^55^. We didn’t find the abundance change of VDAC in Na_2_CO_3_-stressed roots of *P. tenuiflora,* whether it was inhibited needs further investigation. Moreover, the increased Mg^2+^ content in Na_2_CO_3_-stressed *P. tenuiflora* roots and the constant levels in seedlings under 50 mM and 150 mM NaCl^24^ would benefit chlorophyll synthesis, enzyme activation, and the stabilization of nucleotides and nucleic acids to cope with alkali/salt stresses^54^ (Fig. 8C).

In roots, Na^+^ exclusion is a vital strategy to cope with salt and alkali stress. Na^+^ can be exported out of cells by Na^+^/H^+^ antiporter driven by the transmembrane proton electrochemical gradient generated by P-ATPase^56^. In *P. tenuiflora*, a PM Na^+^/H^+^ antiporter encoding gene has been identified as *PtNHA1*^21^. *PtNHA1* was preferentially expressed in roots and up-regulated under 75–300 mM NaCl and 300 mM NaHCO_3_^21^,^27^ (Fig. 8C). *Arabidopsis* over-expressing *PtNHA1* displayed NaCl tolerance with less Na^+^ and more K^+^ accumulations when compared to wild type plants^21^. In addition, alkali response of P-ATPase at gene and protein levels was also studied in other plants. For example, genes encoding P-ATPase isoforms were down-regulated in roots of woody halophyte *T. hispida* under 300 mM NaHCO_3_^13^, but the protein abundance of P-ATPase was increased in roots of glycophyte tomato under 50 mM NaHCO_3_^42^. This implies that different mechanisms of Na^+^ exclusion through P-ATPase under alkali stress might lie between glycophytes and halophytes. In *P. tenuiflora*, although a homologous gene of P-ATPase was Na_2_CO_3_-induced in seedlings^17^, the protein abundance of P-ATPase was decreased in roots under Na_2_CO_3_ stress (Figs 7 and 8C; Table 1). The Na_2_CO_3_-inhibited P-ATPase can lead to low proton driving force in the PM of roots, then reduce Na^+^ efflux through Na^+^/H^+^ antiporters. This probably can be explained by the previous notion that lower Na^+^ accumulation in *P. tenuiflora* is mainly contributed from the restriction of unidirectional Na^+^ influx rather than enhancement of Na^+^ efflux when compared with what happened in wheat seedlings under NaCI stress^2^.

Na^+^ remaining in root cells can be sequestered into vacuoles by vacuolar Na^+^/H^+^ antiporters^8^. In *P. tenuiflora*, a gene encoding vacuolar Na^+^/H^+^ antiporter has been identified as *PutNHX* involved in Na^+^ compartmentalization into vacuole^27^. The expression level of *PutNHX* in *P. tenuiflora* roots under NaHCO_3_ was significantly higher than that under NaCl, indicating that vacuolar Na^+^/H^+^ antiporter may be specifically involved in pH regulation under alkaline conditions^27^ (Fig. 8C). Different from NaCl, NaHCO_3_ and Na_2_CO_3_ stresses generate higher intracellular pH environment, imposing severe damage on plants. Therefore, intracellular pH homeostasis is usually modulated through Na^+^ compartmentalization under alkaline stress. Vacuolar-type H^+^-transporting ATPase (V-ATPase) and vacuolar proton-inorganic pyrophosphatase (V-PPase) can provide proton driven force for vacuolar Na^+^/H^+^ antiporter^57^. It was found that over-expressing the *P. tenuiflora* V-ATPase c subunit (VHA-c) encoding gene *PutVHA-c* in transgenic *Arabidopsis* resulted in better growth phenotypes under salt stress^58^.

In *P. tenuiflora* seedlings, two genes encoding V-ATPase and a gene encoding V-PPase were up-regulated under Na_2_CO_3_ stress^17^ (Fig. 8C). The increased V-ATPase was also found in NaHCO_3_-treated tomato roots^42^, as well as in NaCl-stressed roots of several plants (e.g., rice, wheat, maize, pea (*Pisum sativum*), sugar beet (*Beta vulgaris*), and cucumber (*Cucumis sativus*))^59^. The alkali-/salt-induced *PutNHX, V-ATPase*, and *V-PPase* indicate that the ability of Na^+^ compartmentalization in *P. tenuiflora* is enhanced to cope with stress. More importantly, these proteins also function as H^+^-transporters contributing to the promotion of intracellular pH homeostasis under alkaline conditions. Furthermore, to balance the osmotic pressure in vacuoles resulted from compartmentalized Na^+^, the accumulation of various osmolytes in cytosol is required^8^. In this study, we found soluble sugar content was decreased, while the content of glycine betaine was increased in *P. tenuiflora* roots under Na_2_CO_3_ stress (Fig. 8C). This suggests that the accumulated glycine betaine may play a key role in maintaining cellular osmotic balance in *P. tenuiflora* roots under alkali stress.

### Vesicle trafficking in roots under Na_2_CO_3_

In roots, the dynamics of endomembrane system and vesicle trafficking are very sensitive to ionic and osmotic imbalance resulted from salt/alkaline stress. Previous proteomic studies have found some NaCl-responsive vesicle trafficking-related proteins in roots of various plant species, including annexin, soluble N-ethylmaleimide-sensitive factor attachment protein (SNAP), SNAP receptor, vacuolar-sorting receptor 1, and protein transport protein sec1^59^. These proteins function in vesicle trafficking by regulating the processes of tethering/docking and membrane fusion to enhance salt tolerance^60^,^61^,^62^. In this study, we found more players, such as Na_2_CO_3_-increased Rab1, and Na_2_CO_3_- decreased DRP, VAMP, and MPT (Fig. 8D,F; Table 1), which participate in the trafficking from the ER to the Golgi apparatus, vesicle trafficking, endocytosis/exocytosis, and Pi influx to mitochondrion, respectively^63^,^64^,^65^,^66^. Consistently, transcriptomic analysis revealed that *VAMP* was down-regulated in Na_2_CO_3_-stressed *P. tenuiflora* seedlings^17^ (Fig. 7) and NaHCO_3_-stressed *T. hispida* roots^13^. In addition, *Rab1a* and *Rab1b* in wild soybean roots^12^ and *DRP* in roots of *T. hispida*^13^ were down-regulated under NaHCO_3_. MPT was NaHCO_3_-induced at gene and protein levels in roots of *T. hispida*^13^ and tomato^42^, respectively. Furthermore, *VAMP* and *DRP* displayed diverse expression levels in wild soybean roots under NaHCO_3_^12^. These results indicate that dynamic modulation of cellular transport system is required for maintaining cellular homeostasis under alkaline stress, and the changes of aforementioned players in this system are modulated transiently and dependent on plant species and stress conditions.

### Regulation of Na_2_CO_3_-responsive gene expression, protein processing and destination

Alkali-induced gene expression is regulated by specific chromosome dynamics and transcription factors (Fig. 8E). Proteins involved in gene expression and protein fate were grouped together with strong associations in PPI network (Fig. 6). Among them, histone H2B was decreased, but its chaperone, NAP, was increased in *P. tenuiflora* roots under Na_2_CO_3_ stress, respectively (Table 1). The down-regulated histone H2B gene was also found in *T. hispida* roots under NaHCO_3_ for 48 h^13^. This indicates that dynamic chromosome assembly exists in alkali-stressed roots, which would facilitate chromosome remodeling for alkali-specific gene expression regulation^67^. In our results, transcription factors PURα1 and RRM were increased in Na_2_CO_3_-stressed roots (Table 1). PURα has been proposed to participate in the regulation of *sucrose synthase 1* gene expression in rice^68^, and the NaHCO_3_-induced *PUR*α*1* was found in wild soybean roots^12^. RRM was reported to be involved in almost all post-transcriptional events, especially plastid RNA editing in plants^69^. The alkali-increased PURα1 and RRM may contribute to specific alkali-responsive gene expression. Additionally, eIF3 was increased in *P. tenuiflora* roots under 24 h of Na_2_CO_3_ compared with 12 h of stress (Table 1), and eIF2b was increased under Na_2_CO_3_ stress. This is consistent with up-regulated homologous *eIF3* gene in *P. tenuiflora* seedlings under Na_2_CO_3_^17^ (Fig. 7) and in *T. hispida* roots under NaHCO_3_^13^, implying the enhanced alkali-responsive gene expression.

Previous transcriptomic analysis has shown that the down-regulated genes are often over-represented in alkali-stressed roots. For example, 62.9% of alkali-responsive genes in maize roots under Na_2_CO_3_^11^ and over 70% alkali-responsive genes in roots of wild soybean^12^ and woody halophyte *T. hispida* under NaHCO_3_^13^ were down-regulated. In *P. tenuiflora* seedlings, 69.5% (260 out of 374) and 63.8% (510 out of 799) alkali-responsive genes were down-regulated under 150 mM and 200 mM Na_2_CO_3_ for 12 h, respectively^17^. Consistent with gene expression, our proteomic results presented 57.3% (39 out of 68) alkali-responsive proteins were decreased in *P. tenuiflora* roots under Na_2_CO_3_ stress (Table 1). Among them, 17 ribosomal proteins were alkali-decreased (Table 1). This was consistent with the transcriptomic results that almost all the genes involved in protein synthesis were down-regulated in *P. tenuiflora* seedlings under Na_2_CO_3_ stress^17^ (Fig. 7). This implies that the protein synthesis machinery in *P. tenuiflora* roots is inhibited by Na_2_CO_3_ stress. Similarly, ribosomal proteins also presented NaHCO_3_ and NaCl-decreased abundances in roots of tomato^42^ and *Arabidopsis*^70^. However, in roots of 300 mM NaHCO_3_-stressed *T. hispida*, most genes encoding ribosomal proteins were down-regulated at 12 h, but up-regulated at 48 h^13^. Ribosomal proteins were increased in roots of sugar beet^71^ and cucumber^72^ after 7 days of 50 mM and 500 mM NaCl stress. These results indicate that protein synthesis machinery in roots tends to be inhibited by short-term alkali/salt stress, but it can be activated after a long-term period of stress.

Protein processing in *P. tenuiflora* roots is also affected by Na_2_CO_3_ stress, as shown by the decreased abundances of NAC, CPN60, TCP1, and Hsp90 (Fig. 8E; Table 1). Consistently, NAC in rice roots and *NAC* gene in wild soybean roots were decreased under 150 mM NaCl^73^ and 50 mM NaHCO_3_^12^, respectively. In addition, homologous *CPN60* and *Hsp90* were down-regulated in *P. tenuiflora* seedlings under 150 mM and 200 mM Na_2_CO_3_ for 12 h^17^ (Fig. 7), and Hsp90 was decreased in NaCl-stressed roots of sugar beet^71^ and creeping bentgrass (*Agrostis stolonifera*)^74^. NAC acts as a component of ribosome-associated chaperones. It can associate with ribosome, interact with nascent proteins and protect them from proteolysis, and facilitate their folding^75^. Besides, CPN60 and TCP1 belong to different groups of chaperonin. CPN60 was found in the mitochondrion and plastid, and TCP1 was localized in cytosol, being involved in assisting the folding of newly synthesized and translocated proteins^76^. In addition, Hsp90 functions as a molecular chaperone for assisting protein folding and protein complex formation in many processes, such as signal transduction, cell-cycle control, protein degradation and protein trafficking^76^,^77^. The four proteins (i.e., NAC, CPN60, TCP1, and Hsp90) being decreased in roots implies that the processing/folding of nascent peptides, proteins in different subcellular locations (e.g., mitochondrion, plastid, and cytosol), and protein complexes are all inhibited under salt or alkali stress.

Selective protein degradation in roots is also regulated by Na_2_CO_3_. We found five subunits of 26S proteasome (P26S2, P26S4, P26S6B, P26SLP, and PSA) in *P. tenuiflora* roots were affected by Na_2_CO_3_ stress (Fig. 8E). Some alkali-responsive genes of 26S proteasome subunits were also affected in Na_2_CO_3_-stressed *P. tenuiflora* seedlings^17^ (Fig. 7) and NaHCO_3_-treated wild soybean roots^12^. Moreover, proteomic results showed that AAA+ ATPase, which appeared strong association with proteasome proteins in PPI network (Fig. 6), was increased under Na_2_CO_3_ stress. In addition, MAP was decreased in *P. tenuiflora* roots under Na_2_CO_3_ for 12 h compared with 24 h of treatments, while the *MAP* gene was down-regulated in *P. tenuiflora* seedlings under 200 mM Na_2_CO_3_ for 12 h^17^ (Fig. 7). Considering all these aforementioned results, it becomes evident that protein synthesis and turnover in *P. tenuiflora* roots are inhibited by Na_2_CO_3_. This can account for the reduction of root growth under alkali stress.

### Carbohydrate and energy metabolism was affected under Na_2_CO_3_ stress

Carbohydrate and energy metabolism plays a vital role in root salt/alkali response. It was reported that a number of enzymes involved in glycolysis, TCA cycle, electron transport chain, and pentose phosphate pathway in roots were affected by NaCl stress^59^. In this study, we found that pyruvate kinase (accession number F2CX32) involved in glycolysis was increased in *P. tenuiflora* roots under Na_2_CO_3_ stress (Fig. 8F; Table 1). This correlates well with the previous result that a homologous gene of pyruvate kinase was up-regulated in *P. tenuiflora* seedlings under Na_2_CO_3_^17^ (Fig. 7). Besides, sorbitol dehydrogenase, catalyzing the oxidation of sorbitol to fructose, which can also enter into glycolysis, was increased in *P. tenuiflora* roots under Na_2_CO_3_ (Fig. 8F; Table 1). In addition, dihydrolipoyllysine-residue succinyltransferase, a vital component of 2-oxoglutarate dehydrogenase complex involved in TCA cycle, was increased in roots under Na_2_CO_3_ stress (Fig. 8F; Table 1). Moreover, we found transaldolase, an enzyme of the non-oxidative phase of the pentose phosphate pathway, was increased in *P. tenuiflora* roots under Na_2_CO_3_ (Fig. 8F; Table 1). This is consistent with the Na_2_CO_3_- and NaHCO_3_-induction of homologous genes of transaldolase in *P. tenuiflora* seedlings^17^ (Fig. 7) and wild soybean roots^12^, respectively. These results indicate that glycolysis, TCA cycle, and pentose phosphate pathway are all probably enhanced in alkali-stressed roots to provide energy, carbon skeletons, and NADPH for cellular metabolism.

Besides of these processes, ATP synthase acts as an important enzyme to provide energy for the cells through the synthesis of ATP. A previous study found that *RMtATP6* gene encoding mitochondrial ATP synthase 6 kDa subunit was increased in rice roots under NaCl, NaHCO_3_, and Na_2_CO_3_, respectively^1^. However, in the present study, ATP synthase was decreased in *P. tenuiflora* roots under Na_2_CO_3_ stress (Table 1). This indicates that ATP synthase in *P. tenuiflora* roots is sensitive to alkali stress, leading to the decrease of energy supply through ATP synthase under Na_2_CO_3_ stress.

### Common and specific alkali-responsive strategies between roots and leaves

Plant root functions as the first site for sensing and transducing alkali stress signal. Our Na_2_CO_3_-responsive proteomic studies provided important information for understanding the common and specific alkali-responsive strategies in roots and leaves from *P. tenuiflora^25^.* Nine common alkali-responsive proteins were found in both roots and leaves of *P. tenuiflora* when exposed to Na_2_CO_3_ treatment^25^. Among them, DREPP, eIF, proteasome subunit, and AAA+ ATPase were Na_2_CO_3_-increased in both roots and leaves, indicating that certain processes in signal transduction and proteins synthesis/processing/turnover were usually enhanced in roots and leaves. However, RRM, ribosomal protein, phosphoglycerate kinase, ATP synthase, and aspartate aminotransferase showed different abundance changes between roots and leaves. These diverse protein patterns imply that the processes of energy supplying, gene expression, as well as protein synthesis and amino acid metabolism are modulated in roots and leaves to cope with alkali stress, but different strategies are depended on different organs and various treatment conditions (i.e., alkali concentration and treatment time). Besides of these common proteins in roots and leaves, the physiological and proteomic analyses also highlighted some common pathways, such as Ca^2+^ signal transduction, ROS scavenging system, ion compartmentation, and carbohydrate metabolism, were all induced in roots and leaves under Na_2_CO_3_ stress^25^. At the same time, several specific mechanisms were revealed in roots and leaves^25^. For example, the reduction of light absorption, exudation of salts through stomata, increase of thermal dissipation, and enhance of energy supply were supposed to be employed in leaves^25^, while the enhanced pH modulation was taken as an positive strategy in roots of *P. tenuiflora* to cope with alkali stress.

## Conclusion

**Figure 9:**
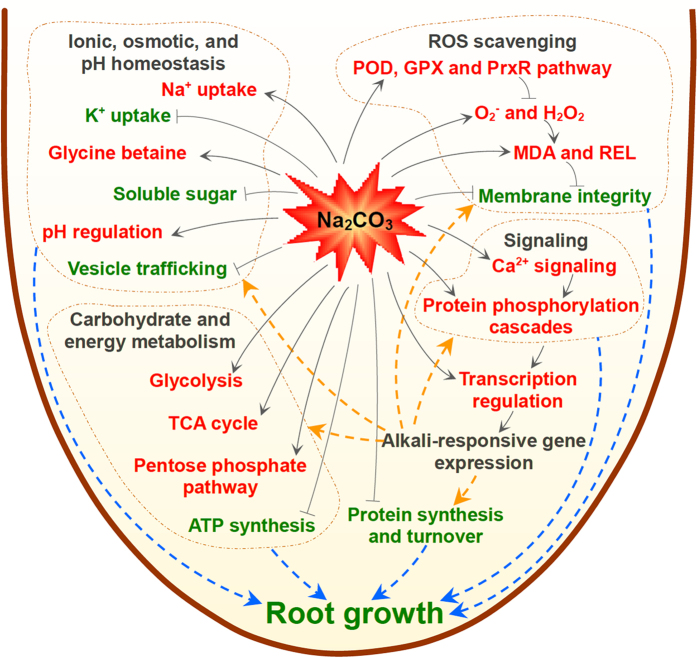
Schematic presentation of systematic Na_2_CO_3_ tolerance mechanisms in roots of *Puccinellia tenuiflora*. Na_2_CO_3_ stress activates the modulation of Na^+^ influx restriction, Na^+^ compartmentalization, H^+^ transportation, glycine betaine accumulation, and vesicle trafficking in roots, which contribute to intracellular pH, ionic, and osmotic homeostasis. In addition, alkali stress leads to ROS burst in roots, resulting in the damages of root cell membrane. To alleviate ROS toxicity, specific ROS scavenging pathways (e.g., POD, GPX, and PrxR pathways) are induced in roots. Na_2_CO_3_ induces glycolysis, TCA cycle, and pentose phosphate pathway, providing energy, carbon skeletons, and NADPH for cellular metabolism in stressed roots. Importantly, Na_2_CO_3_ stress increases the Ca^2+^-mediated signaling pathway, activates the protein phosphorylation cascades, and subsequently triggers alkali-responsive gene expression. However, the protein synthesis, processing and destination are inhibited in roots under Na_2_CO_3_ stress. Solid line with arrow and “T” shape line represent stimulation and inhibition, respectively. The red words and green words indicate Na_2_CO_3_-induced and Na_2_CO_3_-reduced cellular processes, respectively. Dashed lines indicate indirect regulations. Abbreviations: GPX, glutathione peroxidase; MDA, malondialdehyde; POD, peroxidase; PrxR, peroxiredoxin; REL, relative electrolyte leakage; ROS, reactive oxygen species; TCA, tricarboxylic acid.

The signaling and metabolic molecular mechanisms for alkali-tolerance are fine-tuned and sophisticated in roots. Halophytes are supposed to have unique pathways/strategies to cope with alkalinity. In this work, by integrating analysis of the physiological and iTRAQ-based proteomic data, we found multiple Na_2_CO_3_-responsive strategies in *P. tenuiflora* roots (Fig. 9). It mainly includes (1) the activation of Ca^2+^-mediated signaling pathway, (2) specific ROS scavenging pathways (e.g., POD, GPX, and PrxR pathways), (3) modulation of Na^+^ influx restriction, Na^+^ compartmentalization, H^+^-transport, glycine betaine accumulation, and vesicle trafficking, contributing to intracellular pH, ionic and osmotic homeostasis, (4) down-regulation of gene expression, transcription, and protein processing and destination for specific alkali-responsive pathways, as well as (5) induced pathways of glycolysis, TCA and pentose phosphate. These results provide new information and insights into the underlying alkali-responsive mechanism in roots.

## Methods

### Plant growth conditions and Na_2_CO_3_ treatment

Seeds of *Puccinellia tenuiflora* (Turcz.) scribn. et Merr. were sown on pearlite and grown hydroponically in Hoagland solution under fluorescent light (300 μM·m^−2^·s^−1^, 13 h light/11 h dark) at 25 °C and 75% relative humidity in a growth chamber^24^. The nutrient solution was renewed every other day for a stable nutrient supply. Fifty-day-old seedlings were treated with 150 mM and 200 mM Na_2_CO_3_ (pH 11.0) in Hoagland solution for 12 h and 24 h, respectively. After Na_2_CO_3_ treatments, roots of control and Na_2_CO_3_-treated plants were excised, washed gently and briefly in deionized water, and then blotted dry on filter paper. The collected roots were used fresh or immediately frozen in liquid nitrogen and stored at −80 °C. At least three biologically independent replicates for each treatment were collected.

### Root biomass measurement

Root length and Fw were measured immediately after harvesting. Roots were floated on deionized water for 24 h, and then the turgid weight (Tw) was quickly measured. Root Dw was determined after oven-dried at 80 °C for 2 h followed by 60 °C to a constant weight. The RWC was calculated as: RWC = [(Fw-Dw)/(Tw-Dw)] × 100%^78^.

### Ion content analysis

Root tissue was ground into powder after oven-dried at 80 °C for 2 h followed by 60 °C to a constant weight. The powder was digested in nitric acid and perchloric acid solution (5:1, v/v) and incubated at room temperature overnight. The mixture was boiled on an electric stove until the turbid solution became pellucid. After 6 M HCl was added, the solution was diluted to the appropriate concentration with deionized water for ion analysis. The contents of Na^+^, K^+^, Ca^2+^ and Mg^2+^ were assayed using an atomic absorption spectrophotometer AAnalyst 800 (Perkin Elmer, Wellesley, MA, USA) at 589 nm, 766.5 nm, 422.7 nm and 285.2 nm, respectively.

### Total soluble sugar, glycine betaine, MDA and REL measurement

The content of total soluble sugar was determined using a sulfuric acid-anthrone method^79^. For the glycine betaine assay, root tissue was ground with the 60% methanol and 25% chloroform solution. The homogenate was incubated for 24 h, and then centrifuged at 15,000 *g* for 15 min at 20 °C. The supernatant was collected and filtered through a 0.45-μm-pore-size cellulose acetate filter. The filtrate was dried and then resuspended in distilled water. After 0.15% reinecke salt solution was added, the reaction solution was incubated at 4 °C for 2 h. The supernatant was collected after centrifuged at 1,500 *g* for 15 min at 4 °C. After aether was added, the mixed solution was centrifuged at 1,500 *g* for 15 min at 4 °C. The supernatant was collected, dried, and redissolved in 70% acetone. The absorbance was detected under 525 nm using a UV-1800 spectrophotometer (Shimadzu, Tokyo, Japan). The glycine betaine content was calculated from the standard curve. The MDA content and REL were determined according to a previous method^80^.

### ROS measurement and enzyme activity assay

To evaluate the levels of ROS in roots, H_2_O_2_ content and O_2_^−^ generation rate were measured. Root tissue was ground with 0.1% trichloroacetic acid. The homogenate was centrifuged at 15,000 *g* for 15 min at 4 °C and the supernatant was collected for H_2_O_2_ measurement. H_2_O_2_ content was determined spectrophotometrically at 390 nm after reacting with potassium iodide^81^.

To determine O_2_^−^ generation rate and antioxidant enzyme activities, root tissue was ground in extraction buffer containing 50 mM phosphate buffer solution (pH 7.8), 2% polyvinylpyrrolidone-40, and 2 mM AsA (for APX activity assay) at 4 °C. The homogenate was centrifuged at 20,000 *g* for 15 min at 4 °C, and the supernatant was collected for analysis. O_2_^−^ generation rate was measured using a hydroxylamine oxidization method^82^. The activities of SOD, GO, APX, GR, and GST were determined according to our previous methods^24^. The activities of CAT, MDHAR, POD, and DHAR were measured by monitoring H_2_O_2_ consumption at 240 nm, monodehydroascorbate reduction at 340 nm, production of tetraguaiacol at 470 nm, and dehydroascorbate reduction at 265 nm, respectively^83^. Their activities were expressed as the amount of H_2_O_2_ reduced, NADH oxidized, and products of tetraguaiacol and AsA per minute per milligram protein, respectively. GPX activity was determined using a Cellular GPX Assay Kit (Beyotime, Shanghai, China) according to the manufacturer’s instructions. The activity of GPX was expressed as the amount of NADPH oxidized per minute per milligram protein. In all the enzymatic preparations, protein content was determined using the Bradford method^84^.

### Protein extraction for proteomics

Total root protein was extracted from three biological replicate samples for each treatment using a phenol extraction protocol^80^. Proteins were dissolved in a lysis buffer (7 M urea, 2 M thiourea, 4% CHAPS). The protein content was determined using a EZQ Protein Quantitation Kit (Molecular Probes, Eugene, OR, USA) according to the manufacturer’s instructions.

### Trypsin digestion, iTRAQ labeling, and protein fractionation

An aliquot (100 μg) of proteins was precipitated with cold acetone. The pellet was dissolved in 0.5 M triethylammonium bicarbonate (pH 8.5), reduced with 50 mM tris-(2-carboxyethyl)-phosphine, and alkylated with 200 mM methyl methanethiosulfonate (MMTS) using the iTRAQ reagent kit (AB Sciex, Framingham, MA, USA). The proteins were then digested by trypsin (Promega, Madison, WI, USA) and labeled with 8-plex iTRAQ reagents according to the manufacturer’s instructions (AB Sciex, Framingham, MA, USA) using iTRAQ tags 113, 114, 115, 116, and 117 for samples under control condition, 150 mM Na_2_CO_3_ treated for 12 h, 200 mM Na_2_CO_3_ treated for 12 h, 150 mM Na_2_CO_3_ treated for 24 h, and 200 mM Na_2_CO_3_ treated for 24 h, respectively. After labeling, the samples were combined and lyophilized. The peptide mixture was dissolved in 0.1% formic acid and desalted on a Macrospin Vydac Silica C18 column (The Nest Group, Southborough, MA, USA). After desalting, the peptides were dried down and dissolved in strong cation exchange solvent A (25% (v/v) acetonitrile, 10 mM ammonium formate, pH 2.8). The peptides were fractionated on an Agilent high-performance liquid chromatography 1260 infinity system (Agilent Technologies, Palo Alto, CA, USA) with a polysulfoethyl A column (2.1 × 100 mm, 5 μm, 300 Å, PolyLC, Columbia, MD, USA). Peptides were eluted at a flow rate of 200 μl/min with a linear gradient of 0–20% solvent B (25% (v/v) acetonitrile, 500 mM ammonium formate) over 80 min followed by ramping up to 100% solvent B in 5 min and holding for 10 min. The absorbance at 214 nm was monitored, and a total of 13 fractions were collected, lyophilized and dissolved in 0.1% formic acid for LC-MS/MS analysis.

### Mass spectrometry (MS) analysis

An aliquot from each fraction was submitted to a TripleTOF 5600 system (AB Sciex, Framingham, MA, USA) coupled to an Ultra 2D Plus nanoflow ultra-performance LC with a cHiPLC Nanoflex microchip device (Eksigent Technologies, Redwood City, CA, USA). The online trapping, desalting, and analytical separation were conducted using the microfluidic traps and columns packed with ChromXP C18 (3 μm, 120 Å) of the Nanoflex system. Solvent A and B were composed of water/acetonitrile/formic acid (A, 98/2/0.1%; B, 2/98/0.1%). After peptide loading, trapping and desalting were carried out at 2 μL/min for 10 min with 100% solvent A. At a flow rate of 300 nL/min, the analytical separation was established by increasing solvent B from 5% to 10% in 0.1 min, and a linear gradient to 26% solvent B in 60 min. Then the gradient was increased to 50% solvent B in 25 min, kept increasing to 80% solvent B in 1 min, and maintained at 80% solvent B for 4 min. Initial chromatographic condition was restored in 0.1 min and maintained for 10 min. Data were acquired using an ion spray voltage of 2.3 kV, curtain gas of 30, nebulizer gas of 6, and an interface heater temperature of 150 °C. The MS was operated with a resolution of 30,000_fwhm_ for time-of-flight MS scans. For information-dependent acquisition, survey scans were acquired in 250 ms and as many as 30 product ion scans with 100 ms accumulation time were collected if they exceeded a threshold of 150 counts per second (counts/s) and with a 2+ to 5+ charge state. The total cycle time was fixed to 3.3 s. Four time bins were summed for each scan at a pulse frequency value of 11 kHz through monitoring the 40 GHz multichannel detector with four-anode/channel detection. A sweeping collision energy setting of 35 (15 eV) was applied to all precursor ions for collision-induced dissociation. Dynamic exclusion was set for 1/2 of peak width (∼ 18 s), and then the precursor was refreshed off the exclusion list.

### Protein identification and quantification

The MS/MS data were analyzed for protein identification and quantification by searching against a UniProt database (taxonomy *Viridiplantae*, 2, 313, 498 entries, downloaded on 14 May 2013) using ProteinPilot Software 4.5 (AB Sciex, Framingham, MA, USA). The false discovery rate was determined to be 1.0% with the integrated Proteomics System Performance Evaluation Pipeline tool in the ProteinPilot Software. Search parameters included iTRAQ 8-plex quantification, cysteine modified with MMTS, trypsin digestion, an ID focus of biological modifications and amino acid substitutions, thorough searching mode and minimum protein threshold of 95% confidence (unused protein score ≥1.3). To avoid the redundancy when combining proteins from three replicates, the proteins with similar protein family name and amino acid sequence but identified only in one replicate were taken as one unique protein in the final dataset. For protein relative quantification, only MS/MS spectra unique to a particular protein and for which the sum of the signal-to-noise ratio for all of the peak pairs greater than nine were used for quantification (software default settings). For a differentially abundant protein, it had to be quantified with at least three spectra (allowing generation of a *p* value), a *p* value < 0.05, and a ratio fold change ≥1.5 in at least two independent replicates. Only the significant ratios from the replicates were used to calculate the average ratio for the protein^85^.

### Protein classification and PPI analysis

Protein functional classification of the identified proteins were performed manually by searching against the NCBI non-redundant protein database (http://www.ncbi.nlm.nih.gov/) using PSI and PHI-BLAST programs (http://www.ncbi.nlm.nih.gov/BLAST/) for protein functional domain annotation. The biological function of protein was obtained from the KEGG pathway database (http://www.kegg.jp/kegg/), UniProt database (http://www.ebi.uniprot.org/), and the Gene Ontology protein database (http://geneontology.org). Besides, the conservative protein function during salt/alkali tolerance was predicted from previous publications on the salt-/alkali-responsive mechanism in plant root. Finally, by integrative analysis of all the information collected from aforementioned processes, proteins were classified into different categories.

The PPIs were predicted using Search Tool for the Retrieval of Interacting Gene (STRING, version 9.1) (http://string-db.org). The differentially abundant proteins homologs in *Arabidopsis* were found by sequence BLASTing in TAIR database (http://www.arabidopsis.org/Blast/index.jsp). The homologs were subjected to STRING for creating the proteome-scale interaction network. Parameters for species and confidence were “*Arabidopsis thaliana*” and “medium confidence (0.400)”, respectively. STRING analysis was based on “confidence” mode, and disconnected nodes were hidden.

### Correlation analysis of Na_2_CO_3_-responsive proteins and corresponding genes

To find homologous genes, each Na_2_CO_3_-responsive protein sequence was aligned to a cDNA library of *P. tenuiflora* containing 4,982 unigenes^17^ using TBLASTN algorithm (NCBI Blast 2.3.31+). Besides, the genes in cDNA library encoding similar functional domains with Na_2_CO_3_-responsive proteins were also selected for correlation analysis on the basis of functional domain analysis using BLASTP program (http://blast.ncbi.nlm.nih.gov/Blast.cgi). The expression levels of the selected genes under Na_2_CO_3_ and NaCl stresses were evaluated by microarray assay^17^. The correlation of Na_2_CO_3_-responsive proteins and their corresponding genes were analyzed using cluster 3.0 (http://bonsai.hgc.jp/~mdehoon/software/cluster/software.htm).

### Statistical analysis

All results were given as means ± standard deviation of at least three replicates. The data were subjected to one-way analysis of variance using SPSS 17.0 software (SPSS, Chicago, IL, USA). A *p* value smaller than 0.05 was considered to be statistically significant.

## Additional Information

**How to cite this article**: Zhao, Q. *et al.* Na_2_CO_3_-responsive mechanisms in halophyte *Puccinellia tenuiflora* roots revealed by physiological and proteomic analyses. *Sci. Rep.* **6**, 32717; doi: 10.1038/srep32717 (2016).

## Supplementary Material

Supplementary Information

## Figures and Tables

**Table 1:** Na_2_CO_3_-responsive proteins in roots of *Puccinellia tenuiflora* identified by iTRAQ-based proteomic analysis.

Accession No.^a^	Protein name^b^	Species^c^	Mw (Da)^d^	pI^e^	Relative protein abundance^f^			
					150 mM, 12 h	200 mM, 12 h	150 mM, 24 h	200 mM, 24 h
	**Signaling (6)**							
D2EDB7	Salt-stress root protein, containing pfam05558 developmentally regulated plasma membrane polypeptide domains* (DREPP)	*Lolium perenne*	22,106	4.97	8.015 ± 3.453*	8.810 ± 2.054*	12.880 ± 1.474*	9.304 ± 1.366*
Q5MCL9	Calreticulin-like protein (CRT)	*Triticum aestivum*	47,204	4.49	5.203 ± 2.886*	3.900 ± 2.185*	7.697 ± 5.203*	6.204 ± 3.544*
B4FAK8	Putative uncharacterized protein, containing pfam00262 calreticulin domain* (CRT)	*Zea mays*	60,246	4.70	0.748 ± 0.097	0.526 ± 0.149*	2.110 ± 0.110	0.518 ± 0.037
F2E2L5	Predicted protein, containing cd00051 calcium binding motif, calmodulin* (CaM)	*Hordeum vulgare* var*. distichum*	16,670	4.89	0.997 ± 0.071	1.043 ± 0.061	0.517 ± 0.303	0.458 ± 0.286*
Q6KCK6	Calcium-dependent protein kinase (CDPK)	*T. aestivum*	58,408	5.79	2.555 ± 1.201	2.764 ± 1.087	3.321 ± 1.145*	3.351 ± 1.378*
F2CQQ4	Serine/threonine-protein phosphatase, containing cd07414 protein phosphate type 1 and keltch like domain* (STPP)	*H. vulgare* var*. distichum*	36,177	5.19	1.316 ± 0.339	1.888 ± 0.569	2.331 ± 0.527*	2.506 ± 0.970
	**ROS scavenging (2)**							
F2DTT4	Predicted protein, 2-Cys peroxiredoxin* (PrxR)	*H. vulgare* var*. distichum*	28,230	6.33	2.128 ± 0.575*	2.043 ± 0.729	3.282 ± 0.887*	2.680 ± 0.724*
Q6UQ06	Cytosolic glutathione reductase (GR)	*Triticum. monococcum*	53,015	5.93	2.065 ± 0.440	2.118 ± 0.492	2.580 ± 0.318*	3.445 ± 0.358*
	**Transportation (5)**							
Q5PSM6	Plasma membrane H^+^-ATPase (P-ATPase)	*T. aestivum*	104,661	6.58	0.251 ± 0.024*	0.163 ± 0.018*	0.185 ± 0.026*	0.289 ± 0.043*
F2CRB3	Predicted protein, containing cd01869 Rab1 domain*	*H. vulgare var. distichum*	22,503	5.14	2.100 ± 0.440	1.893 ± 0.588	2.712 ± 0.686*	2.827 ± 1.043*
C5XQM5	Putative uncharacterized protein Sb03g040890, homologue of vesicle-associated membrane protein family protein* (VAMP)	*Sorghum bicolor*	39,780	9.86	0.438 ± 0.057*	0.654 ± 0.119	0.318 ± 0.068	0.327 ± 0.023
Q0DG31	Os05g0556100 protein, dynamin-related protein* (DRP)	*Oryza sativa* subsp*. japonica*	68,683	7.65	0.728 ± 0.005	0.662 ± 0.052	0.345 ± 0.040*	0.525 ± 0.000
A8TU59	Mitochondrial phosphate transporter (MPT)	*Paeonia suffruticosa*	39,853	9.31	0.194 ± 0.005	0.213 ± 0.000	0.105 ± 0.005*	0.106 ± 0.005*
	**Chromosome assembly and transcription (4)**							
F2E328	Histone H2B	*H. vulgare* var*. distichum*	16,236	10.02	0.215 ± 0.009*	0.471 ± 0.035*	0.239 ± 0.018*	0.194 ± 0.043*
F2DVK7	Predicted protein, nucleosome assembly protein* (NAP)	*H. vulgare* var*. distichum*	42,009	4.32	3.057 ± 2.082	2.606 ± 1.402	4.897 ± 4.205*	2.949 ± 1.780
B4FYX0	Putative uncharacterized protein, transcription factor purine-rich alpha 1* (PURα1)	*Z. mays*	33,488	5.72	2.100 ± 0.749	2.189 ± 0.728	3.530 ± 1.472*	2.249 ± 0.898
F2D3D5	Predicted protein, containing cd00590 RNA recognition motif* (RRM)	*H. vulgare* var*. distichum*	41,295	5.82	1.319 ± 0.069	1.441 ± 0.224	2.177 ± 0.506*	1.528 ± 0.000
	**Protein synthesis (19)**							
F2CQY1	Predicted protein, 40S ribosomal protein S3* (RPS3)	*H. vulgare* var*. distichum*	25,373	9.55	0.551 ± 0.100*	0.413 ± 0.000*	0.312 ± 0.085*	0.307 ± 0.055*
F2DIR3	Predicted protein, 40S ribosomal protein S4* (RPS4)	*H. vulgare* var*. distichum*	29,949	10.15	0.164 ± 0.025*	0.255 ± 0.111*	0.073 ± 0.024*	0.143 ± 0.003*
F2D448	Predicted protein, containing pfam00333 ribosomal protein S5 domain* (RPS5)	*H. vulgare* var*. distichum*	30,341	10.18	0.240 ± 0.083*	0.297 ± 0.108*	0.110 ± 0.115*	0.175 ± 0.109*
B4FKA4	Putative uncharacterized protein, 40S ribosomal protein S14* (RPS14)	*Z. mays*	16,363	10.56	0.306 ± 0.115	0.324 ± 0.110*	0.256 ± 0.114	0.307 ± 0.155
D7KHV6	40S ribosomal protein S15a (RPS15a)	*Arabidopsis lyrata* subsp*. lyrata*	14,804	9.89	0.714 ± 0.134	0.708 ± 0.092	0.395 ± 0.107*	0.307 ± 0.231*
F2E598	Predicted protein, containing pfam01090 ribosomal protein S19 domain* (RPS19)	*H. vulgare* var*. distichum*	17,084	9.89	0.438 ± 0.062*	0.599 ± 0.099	0.313 ± 0.047*	0.277 ± 0.072*
Q6V959	Ribosomal protein L3 (RPL3)	*T. aestivum*	44,592	10.07	0.139 ± 0.016*	0.277 ± 0.080	0.141 ± 0.063*	0.255 ± 0.099
Q0D868	Os07g0180900 protein, containing PRK04042 ribosomal protein L4* (RPL4)	*O. sativa* subsp*. japonica*	46,694	10.64	0.145 ± 0.017*	0.287 ± 0.034*	0.071 ± 0.032*	0.162 ± 0.053*
F2DAK3	Predicted protein, 60S ribosomal protein L6* (RPL6)	*H. vulgare* var*. distichum*	24,372	10.10	0.462 ± 0.042*	0.494 ± 0.108*	0.321 ± 0.130*	0.174 ± 0.044*
F2E0C0	Predicted protein, 60S ribosomal protein L7* (RPL7)	*H. vulgare* var*. distichum*	28,287	10.03	0.220 ± 0.061*	0.350 ± 0.131*	0.100 ± 0.026*	0.157 ± 0.040*
F2DE13	Predicted protein, 60S ribosomal protein L7a* (RPL7a)	*H. vulgare* var*. distichum*	29,409	10.34	0.111 ± 0.005	0.208 ± 0.039*	0.106 ± 0.021*	0.164 ± 0.030*
F2CT73	Predicted protein, 60S ribosomal protein L8* (RPL8)	*H. vulgare* var*. distichum*	28,191	11.08	0.306 ± 0.105*	0.444 ± 0.224*	0.184 ± 0.152*	0.181 ± 0.016*
F2DVU2	60S ribosomal protein L13 (RPL13)	*H. vulgare* var*. distichum*	24,129	10.91	0.171 ± 0.051*	0.217 ± 0.027*	0.090 ± 0.004*	0.176 ± 0.060*
Q5I7L1	Ribosomal protein L13a (RPL13a)	*T. aestivum*	23,530	10.39	0.290 ± 0.206*	0.369 ± 0.205*	0.197 ± 0.222*	0.254 ± 0.207*
Q9AXS0	Ribosomal protein L17-1 (RPL17-1)	*Poa secunda*	19,564	10.25	0.338 ± 0.034	0.410 ± 0.100	0.128 ± 0.021*	0.252 ± 0.037
F2EAX5	Predicted protein, 60S ribosomal protein L22* (RPL22)	*H. vulgare* var*. distichum*	14,375	9.56	0.270 ± 0.037*	0.243 ± 0.116*	0.257 ± 0.040*	0.156 ± 0.097*
Q07760	60S ribosomal protein L23 (RPL23)	*Nicotiana tabacum*	14,988	10.48	0.275 ± 0.038	0.337 ± 0.042	0.200 ± 0.066*	0.224 ± 0.073*
F2CTT6	Predicted protein, containing COG0182 translation initiation factor 2b subunit domain* (eIF2b)	*H. vulgare* var*. distichum*	38,573	5.56	4.924 ± 0.256*	2.238 ± 1.266	5.062 ± 0.264*	5.961 ± 1.536*
F2CS01	Predicted protein, eukaryotic initiation factor 3 subunit* (eIF3)	*H. vulgare* var*. distichum*	83,367	5.03	1.854 ± 0.384	1.517 ± 0.187	2.846 ± 0.449*	2.466 ± 0.032*
	**Protein processing (4)**							
A9U4U1	Predicted protein, nascent polypeptide-associated complex subunit alpha-like protein-like* (NAC)	*Physcomitrella patens* subsp*. patens*	21,604	4.35	0.275 ± 0.264*	0.247 ± 0.274*	0.766 ± 0.149	0.327 ± 0.267*
F2EE28	Predicted protein, chaperonin 60* (CPN60)	*H. vulgare* var*. distichum*	61,033	5.45	0.491 ± 0.107*	0.446 ± 0.121*	0.696 ± 0.221	0.423 ± 0.125*
F2DRC5	Predicted protein, T-complex protein 1 subunit beta* (TCP1)	*H. vulgare var. distichum*	57,337	5.63	0.570 ± 0.228	0.679 ± 0.132	0.327 ± 0.055*	0.515 ± 0.020
Q7XJ80	Cytosolic heat shock protein 90 (Hsp90)	*H. vulgare*	80,419	4.95	0.160 ± 0.056*	0.077 ± 0.009*	0.342 ± 0.122*	0.147 ± 0.096*
	**Protein degradation (7)**							
F2E7G1	Predicted protein, 26S protease regulatory subunit 4* (P26S4)	*H. vulgare* var*. distichum*	49,686	5.90	0.586 ± 0.015*	0.714 ± 0.017	0.357 ± 0.117*	0.556 ± 0.121
F2D121	Predicted protein, 26S protease regulatory subunit 6B* (P26S6B)	*H. vulgare* var*. distichum*	45,685	5.74	0.639 ± 0.189	0.587 ± 0.053	0.237 ± 0.147*	0.426 ± 0.112*
D3G8A3	26S protease regulatory subunit-like protein (P26SLP)	*L. perenne*	48,018	4.84	0.649 ± 0.017	0.617 ± 0.004*	0.451 ± 0.059*	0.460 ± 0.092*
F2DQ10	Predicted protein, 26S proteasome regulatory subunit S2* (P26S2)	*H. vulgare* var*. distichum*	98,121	5.05	1.652 ± 0.255	1.797 ± 0.236	2.013 ± 0.175	2.517 ± 0.202*
Q6H852	Proteasome subunit alpha type (PSA)	*O. sativa* subsp*. japonica*	25,844	5.38	2.885 ± 0.996*	2.288 ± 0.761*	3.472 ± 1.378*	2.515 ± 0.819*
Q941B7	At2g39730/T5I7.3, containing pfam00004 ATPase family associated with various cellular activities domain* (AAA+ ATPase)	*Arabidopsis thaliana*	52,039	5.69	1.538 ± 1.312	2.900 ± 1.528*	8.982 ± 5.673*	11.663 ± 6.928*
E0A9F0	Methionine aminopeptidase (MAP)	*H. vulgare*	43,373	6.58	0.805 ± 0.050	0.440 ± 0.100*	0.501 ± 0.000*	0.657 ± 0.07**0**
	**Carbohydrate and energy metabolism (7)**							
P12783	Phosphoglycerate kinase, cytosolic (PGK)	*T. aestivum*	42,121	5.64	0.671 ± 0.104	0.652 ± 0.093	0.185 ± 0.103*	0.444 ± 0.081
F2CS51	Pyruvate kinase (PK)	*H. vulgare* var*. distichum*	55,453	7.50	1.366 ± 0.151	1.092 ± 0.021	1.661 ± 0.108	1.907 ± 0.099*
F2CX32	Pyruvate kinase (PK)	*H. vulgare* var*. distichum*	57,436	6.48	1.038 ± 0.027	1.128 ± 0.073	1.692 ± 0.088*	1.872 ± 0.097*
B6TRW8	Dihydrolipoyllysine-residue succinyltransferase component of 2-oxoglutarate dehydrogenase complex (DLST)	*Z. mays*	48,775	8.95	2.043 ± 0.864	1.660 ± 0.322	2.754 ± 0.376*	2.030 ± 0.649
Q84ZL6	Os08g0154300 protein, containing cd00957 transaldolase domain* (TA)	*O. sativa* subsp*. japonica*	43,019	5.17	2.309 ± 0.739	2.139 ± 0.305	3.391 ± 1.210*	3.136 ± 1.042
F2CYT1	Predicted protein, sorbitol dehydrogenase* (SDH)	*H. vulgare* var*. distichum*	38,905	6.27	1.517 ± 0.138	1.706 ± 0.022	1.915 ± 0.062*	1.928 ± 0.201*
F2CWQ6	ATP synthase gamma chain	*H. vulgare* var*. distichum*	35,424	9.39	0.640 ± 0.004	0.662 ± 0.052	0.377 ± 0.051	0.302 ± 0.037*
	**Amino acid metabolism (5)**							
F2DWA1	Chorismate synthase (CS)	*H. vulgare* var*. distichum*	54,896	8.22	0.512 ± 0.047	0.363 ± 0.066	0.277 ± 0.117*	0.329 ± 0.053
C5IW60	Glutamine synthetase (GS)	*L. perenne*	38,762	5.58	0.504 ± 0.078	0.249 ± 0.121*	0.180 ± 0.092	0.283 ± 0.051*
Q25C96	Aspartate aminotransferase (AAT)	*H. vulgare*	45,173	5.75	0.986 ± 0.054	0.523 ± 0.031*	0.363 ± 0.088*	0.650 ± 0.088
F2D9P4	Predicted protein, *O*-acetylserine (thiol) lyase of cysteine synthase complex* (CysS)	*H. vulgare* var*. distichum*	37,137	5.38	1.440 ± 0.206	1.343 ± 0.183	2.301 ± 0.015*	2.341 ± 0.274
Q5EI64	Pyrroline-5-carboxylate reductase (P5CR)	*T. aestivum*	29,353	8.88	2.704 ± 0.035	1.846 ± 0.060	4.131 ± 0.054*	3.183 ± 0.269*
	**Fatty acid metabolism (3)**							
D2KZ12	3-ketoacyl-CoA thiolase-like protein (KCT)	*T. aestivum*	47,925	8.21	0.606 ± 0.186	0.540 ± 0.215	0.446 ± 0.112*	0.628 ± 0.146
A2WNV6	Putative uncharacterized protein, ATP citrate lyase* (ACL)	*O. sativa* subsp*. indica*	66,069	7.57	0.499 ± 0.016*	0.520 ± 0.071	0.453 ± 0.000*	0.545 ± 0.043*
F2E3J2	Predicted protein, leukotriene A4 hydrolase* (LTA4H)	*H. vulgare* var*. distichum*	67,816	4.99	0.692 ± 0.094	0.664 ± 0.133	0.477 ± 0.117*	0.596 ± 0.066
	**Other metabolisms (10)**							
Q40062	2′-deoxymugineic-acid 2′-dioxygenase (IDS3)	*H. vulgare*	37,732	5.94	0.506 ± 0.121	0.396 ± 0.046	0.128 ± 0.049*	0.363 ± 0.024
F2DIA8	Predicted protein, containing pfam00150 cellulase domain*	*H. vulgare* var*. distichum*	117,787	5.57	1.289 ± 0.166	1.370 ± 0.308	1.924 ± 0.466*	1.552 ± 0.348
F2DIZ2	Predicted protein, coproporphyrinogen III oxidase* (CPOX)	*H. vulgare* var*. distichum*	43,446	7.05	2.270 ± 0.309	1.971 ± 0.307	2.646 ± 0.412*	2.292 ± 0.445
F2CSU5	N-acetyl-gamma-glutamyl-phosphate reductase (AGPR)	*H. vulgare* var*. distichum*	44,838	8.55	2.120 ± 0.425	2.033 ± 0.395	2.496 ± 0.548*	2.192 ± 0.355*
C5WVL6	Putative uncharacterized protein Sb01g031870, containing PLN02343 allene oxide cyclase domain* (AOC)	*S. bicolor*	29,368	9.45	2.607 ± 0.506	3.049 ± 0.919	4.135 ± 0.617*	3.605 ± 0.328
F2DUQ7	Predicted protein, containing cd04727 pyridoxal 5′-phosphate synthase domain* (PdxS)	*H. vulgare* var*. distichum*	33,247	6.60	1.767 ± 0.255	1.908 ± 0.372	2.781 ± 0.399*	3.257 ± 1.182*
F2E2V8	Predicted protein, containing cd08936 peroxisomal carbonyl reductase like, classical SDR domain* (CR)	*H. vulgare* var*. distichum*	26,780	8.42	1.700 ± 0.122	1.649 ± 0.172	2.323 ± 0.076*	2.103 ± 0.178
F2DZ92	Predicted protein, containing cd07572 nitrilase domain*	*H. vulgare* var*. distichum*	33,407	5.82	2.732 ± 0.561*	2.140 ± 0.468*	3.945 ± 0.778*	4.018 ± 0.857*
F2D9Z5	Predicted protein, containing smart00835 cupin domain*	*H. vulgare* var*. distichum*	38,148	5.65	1.571 ± 0.082	1.684 ± 0.077	2.188 ± 0.028*	1.655 ± 0.140
F2CT63	Predicted protein, containing cd04899 C-terminal ACT domains of the bacterial signal-transducing uridylyltransferase/uridylyl-removing enzyme* (UUR)	*H. vulgare* var*. distichum*	33,923	5.92	2.114 ± 0.337*	1.136 ± 0.138	1.598 ± 0.254	1.479 ± 0.172

^a^Database accession numbers from UniProt. ^*b*^The names and functional categories of the proteins identified by iTRAQ-based proteomics analysis. Protein names marked with an asterisk (*) have been edited by us according to functional domain annotations from NCBI non-redundant protein database. The abbreviations for the protein names are indicated in the bracket after protein names. ^*c*^The plant species that the peptides matched from. ^*d,e*^Theoretical mass (Da) (d) and pI (e) of identified proteins. ^*f*^Relative protein abundances under 150 mM Na_2_CO_3_ for 12 h, 200 mM Na_2_CO_3_ for 12 h, 150 mM Na_2_CO_3_ for 24 h, and 200 mM Na_2_CO_3_ for 24 h compared with control condition, respectively. Most of the protein abundance changes were compared with control condition, but the abundance change of PK (Accession No. F2CS51) was compared with 150 mM Na_2_CO_3_ for 12 h, eIF3 and PGK were compared with 200 mM Na_2_CO_3_ for 12 h, and MAP was compared with 150 mM Na_2_CO_3_ for 24 h. The ratios were presented as means ± standard deviation. The asterisks indicate significant differences (*p* < 0.05).
