# Supplementary material for: Na2CO3-responsive mechanisms in halophyte Puccinellia tenuiflora roots revealed by physiological and proteomic analyses
Source: Sci Rep. 2016 Sep 6;6:32717. doi: 10.1038/srep32717 (PMC5011731; doi:10.1038/srep32717)
Supplement: Supplementary Information [file srep32717-s1.pdf]

Na_2CO_3-responsive mechanisms in halophyte *Puccinellia tenuiflora* roots revealed by physiological and proteomic analyses

Qi Zhao[1,2], Jinwei Suo[2], Sixue Chen[3], Yudan Jin[2], Xiaolin Ma[2], Zepeng Yin[2], Yuhong Zhang[1], Tai Wang[4], Ji Luo[5], Wenhai Jin[5], Xia Zhang[4], Zhiqiang Zhou[1,#] & Shaojun Dai[1,2,*]

[1]Key Laboratory of Forest Plant Ecology, Ministry of Education, Northeast Forestry University, Harbin 150040, China;

[2]Alkali Soil Natural Environmental Science Center, Northeast Forestry University, Key Laboratory of Saline-alkali Vegetation Ecology Restoration in Oil Field, Ministry of Education, Harbin 150040, China;

[3]Department of Biology, Genetics Institute, Plant Molecular and Cellular Biology Program, Interdisciplinary Center for Biotechnology Research, University of Florida, Gainesville, FL 32610, USA;

[4]Institute of Botany, Chinese Academy of Sciences, Beijing 100093, China;

[5]AB Sciex Asia Pacific Application Support Center, Shanghai 200233, China

***Correspondence:** Professor Shaojun Dai, Northeast Forestry University, Harbin 150040, China

Tel: 86-451-82192237; **Fax:** 86-451-82192237

E-mail: daishaojun@hotmail.com

[#]Co-corresponding author Tel: 86-451-82190338; **Fax:** 86-451-82190338

E-mail: zhiqiangzhou@hotmail.com

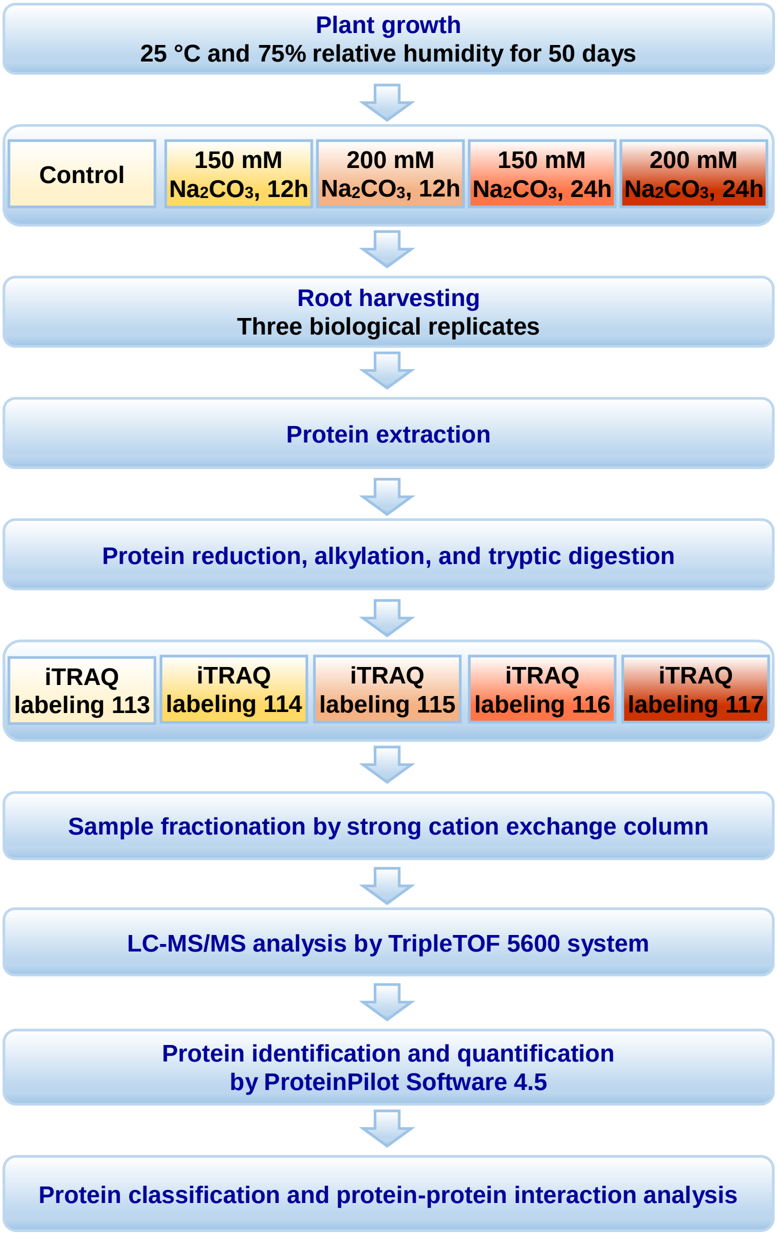

Supplementary Figure S1. iTRAQ-based proteomics strategy for analysis of Na_2CO_3-responsive proteins in *Puccinellia tenuiflora* roots

```
gi|30679935|AtCPK6       1 MGNSCRGSFKDKIY---EGNHSRPEENSKSTTTTVSSVHSPTTDQDFSKQNTNPALVIPV
gi|10944296|OsCDPK7      1 MGNACGGSLRSKYLSFKQTASQRHDTDDNNNAAAADSPKKPSRPPAAAKTDDHPVSASAP
Contig720|PtCDPK         1 ------------------------------------------------------------
consensus                1 ... . .. . .          . .           . . ..      .    .

gi|30679935|AtCPK6      58 KEPIMRRNVDNQSYYVLGHKTPNIRDLYTLSRKLGQGQFGTTYLCTDIATGVDYACKSIS
gi|10944296|OsCDPK7     61 AAAMRRGQAPADLGSVLGHPTPNLRDLYAMGRKLGQGQFGTTYLCTELSTGVDYACKSIS
Contig720|PtCDPK         1 ------------------------------------------------------------
consensus               61    . . .       .... ........ . ................. ...........

gi|30679935|AtCPK6     118 KRKLISKEDVEDVRREIQIMHHLAGHKNIVTIKGAYEDPLYVHIVMELCAGGELFDRIIH
gi|10944296|OsCDPK7    121 KRKLITKEDIEDVRREIQIMHHLSGHKNVVAIKGAYEDQLYVHIVMELCAGGELFDRIIQ
Contig720|PtCDPK         1 --------------------GHLTGHRNIVELHGAYEDRHSVNLIMELCEGGELFDRIIA
consensus              121 .....................**.**.*.* ..***** ..*...****.*********

gi|30679935|AtCPK6     178 RGHYSERKAAELTKIIVGVVEACHSLGVMHRDLKPENFLLVNKDDDFSLKAIDFGLSVFF
gi|10944296|OsCDPK7    181 RGHYSERKAAELTRIIVGVVEACHSLGVMHRDLKPENFLLANKDDDLSLKAIDFGLSVFF
Contig720|PtCDPK        41 RGHYSERAAAALCREIVSVVHSCHSMGVFHRDLKPENFLFLNDKEDSPLKATDFGLSVFF
consensus              181 *******.**.*...**.**..***.**.**********. *...* .***.********

gi|30679935|AtCPK6     238 KPGQIFKDVVGSPYYVAPEVLLKHYGPEADVWTAGVILYILLSGVPPFWAETQQGIFDAV
gi|10944296|OsCDPK7    241 KPGQTFTDVVGSPYYVAPEVLLKHYGPEADVWTAGVILYILLSGVPPFWAETQQGIFDAV
Contig720|PtCDPK       101 KHGEQFKDLVGSAYYVAPEVLKRHYGAEADIWSAGIILYILLSGVPP-------------
consensus              241 *.*. *.*.***.********..***.***.*.**.***********.............

gi|30679935|AtCPK6     298 LKGYIDFDTDPWPVISDSAKDLIRKMLCSSPSERLTAHEVLRHPWICENGVAPDRALDPA
gi|10944296|OsCDPK7    301 LKGFIDFDSDPWPVISESAKDLITKMLNPRPKERLTAHEVLCHPWIRDHGVAPDRPLDPA
Contig720|PtCDPK       148 ------------------------------------------------------------
consensus              301 ....................... ...   . ......... .... . ...... ....

gi|30679935|AtCPK6     358 VLSRLKQFSAMNKLKKMALKVIAESLSEEEIAGLRAMFEAMDTDNSGAITFDELKAGLRR
gi|10944296|OsCDPK7    361 VLSRIKQFSAMNKLKKMALRVIAESLSEEEIAGLKEMFQTMDADNSGAITYDELKEGLRK
Contig720|PtCDPK       148 ------------------------------------------------------------
consensus              361 ................................... ..  .. ............ ....

gi|30679935|AtCPK6     418 YGSTLKDTEIRDLMEAADVDNSGTIDYSEFIAATIHLNKLEREEHLVSAFQYFDKDGSGY
gi|10944296|OsCDPK7    421 YGSTLKDTEIRDLMDAADIDNSGTIDYIEFIAATLHLNKLEREEHLVAAFSYFDKDGSGY
Contig720|PtCDPK       148 FGQTLR-------------------------MAYLRLSCLARISHLTLGLQYL-------
consensus              421 .*.**...................... ....*...*..*.*..**. ...*........

gi|30679935|AtCPK6     478 ITIDELQQSCIEHGMTDVFLEDIIKEVDQDNDGRIDYEEFVAMMQKGNAGVGRRTMKNSL
gi|10944296|OsCDPK7    481 ITVDELQQACKEHNMPDAFLDDVINEADQDNDGRIDYGEFVAMMTKGNMGVGRRTMRNSL
Contig720|PtCDPK       176 ------------------------------------------MAQK----IWR-------
consensus              481 ........ . .. . . ...... . .......... ....*..*.. ...*.......

gi|30679935|AtCPK6     538 NISMRDV----
gi|10944296|OsCDPK7    541 NISMRDAPGAL
Contig720|PtCDPK           -----------
consensus              541 ......
```

Supplementary Figure S2. Multiple amino acid sequence alignment of calcium-dependent protein kinases from Arabidopsis (AtCPK6), rice (OsCDPK7), and

***Puccinellia tenuiflora* (PtCDPK).** The Gi numbers of AtCPK6 and OsCDPK7, and encoding unigene name of PtCDPK were listed on the left side. Completely conserved residues among all three proteins were presented in red fonts and marked with asterisks in the consensus line. The dot indicates conserved region between two proteins. Conserved residues between PtCDPK and AtCPK6/OsCDPK7 were presented in blue fonts.
